# Supplementary material for: Health beliefs and health seeking behavior towards lymphatic filariasis morbidity management and disability prevention services in Luangwa District, Zambia: Community and provider perspectives
Source: PLoS Negl Trop Dis. 2021 Feb 22;15(2):e0009075. doi: 10.1371/journal.pntd.0009075 (PMC7932505; doi:10.1371/journal.pntd.0009075)
Supplement: S1 Data collection tools — (DOCX) [file pntd.0009075.s001.docx]

**COMMUNITY LEADER FGD GUIDE.**

Date: Time:

Interviewer: Location:

Village: Health Facility/District:

| **No.** | **Question** |
| --- | --- |
| **SECTION 1: SERVICE UTILIZATION** | |
| 1.1 | What do most community members believe is the cause of these LF conditions? |
| 1.2 | How do most people manage their condition? (Probe on)   1. Visit traditional healers 2. Visit a pharmacy for supplies and medications 3. Visit a health facility 4. Take of themselves from home |
| 1.3 | What factors prevent people with morbidity associated with LF from getting the healthcare they need? (Probe on)   1. Stigma-how they got the disease 2. Stigma-Shame about having the disease 3. Not knowing how serious their condition is 4. Feeling like there is nothing they can do about their condition 5. Transport 6. Facility wait time 7. Provider interactions 8. Money to pay for services 9. Mobility |
| 1.4 | Do people with these LF conditions receive help from other community members or family members? If no, why? (Probe on) *existing social support groups*...:   1. Help with things like cooking, fetching water, moving around, getting to the health facility or pharmacy, etc |
| 1.5 | What recommendations do you have for improving access to morbidity associated with LF services for those affected by LF?   1. Community level recommendations; community health workers 2. Facility level recommendations 3. Transport related recommendations 4. Financial related recommendations |

**HEALTH PROVIDERS/COMMUNITY HEALTH WORKERS IDI GUIDE.**

Date: Time:

Interviewer: Health provider ID:

Village: Health Facility/Post:

Sex: Age:

Education level: Length of stay in the area:

Position: Number of years practicing:

| **No.** | **Question** | **Code** | **Response** |
| --- | --- | --- | --- |
| **SECTION 1: TRAINING ON MMDP** | | | |
| 1.1 | Have you ever been trained or retrained for lymphoedema/ hydrocele management?  No  Yes  Don’t know  Refused | 0  1  2  3 |  |
| 1.2 | Have you ever been trained or retrained for lymphoedema/ hydrocele management in the past 2 years?  No  Yes  Don’t know  Refused | 1  2  3  4 |  |
| **SECTION 2: MORBIDITY MANAGEMENT** | | | |
| **Please read the following questions to the interviewee. As they respond do not read the answers, ask them to be specific, encourage “anything else?” Until nothing further is mentioned and check all that apply.** | | | |
| 2.1 | Please describe for me the signs and symptoms of lymphedema (Probe for)   1. Swelling (reversible at night) 2. Swelling (irreversible) 3. Skin folds (shallow or deep) 4. Knobs on the skin 5. Mossy lesions (i.e. small elongated or rounded growths) 6. Inability to perform daily activities or care for self 7. Acute attacks / adenolymphangitis 8. Wounds or entry lesions 9. Other; specify: __________________________ 10. Don't know any signs/symptoms of lymphedema | | |
| 2.2 | Please describe for me the signs and symptoms of hydrocele? (Probe for). | | |
| 2.3 | Please describe for me the signs and symptoms of an acute attack (ADL, also known as <local word for ADL>)? (Probe for)   1. Redness of limb 2. Warmth of limb 3. Increased swelling of limb 4. Painful limb 5. Fever 6. Headache 7. Chills 8. Nausea/vomiting 9. Other; specify: _____________________________ 10. Don’t know any signs and symptoms of an acute attack | | |
| 2.4 | Please describe for me all of the strategies you would teach a lymphedema patient for preventing the progression of lymphedema and preventing acute attacks (ADL, also known as <local word for ADL>)? (Probe for)   1. Hygiene / Washing and drying of affected limb 2. Wound care / care of entry lesions 3. Elevation 4. Exercise 5. Shoe use 6. Prophylactic creams 7. Prophylactic systemic antibiotics 8. Instruct patients to avoid harmful behaviors (e.g. scarification and fumigation) 9. Other; specify: _____________________________ 10. Don’t know any lymphedema management techniques | | |
| 2.5 | Please describe for me all of the management strategies you could use to treat a patient who is having an acute attack (ADL, also known as <local word for ADL>)? (Probe for)   1. Cool leg in bucket of cool water or using a cold compress 2. Analgesic or anti-inflammatory medications 3. Topical antibiotics 4. Oral antibiotics 5. Injectable antibiotics 6. Rest 7. Elevation 8. Provide fluids 9. Advise patient to avoid exercises for duration of acute attack 10. Other; specify: ___________________   Don’t know any acute attack treatments | | |
| **SECTION 3: SERVICE PROVISION** | | | |
| 3.1 | What services are available for?   1. Lymphoedema management? 2. Hydrocele management | | |
| 3.2 | How would describe accessibility to?   1. Hydrocele services 2. Lymphedema services | | |
| 3.3 | What factors influence access to services? | | |
| 3.4 | In your opinion, why don’t patients seek services at the facility? (Probe for)   1. Stigma 2. Mobility 3. Transport 4. Paying for services | | |
| 3.5 | Do you conduct outreach activities with regards to hydrocele and Lymphedema management? If yes, how are the activities conducted?   1. Do you provide LF services with other diseases? If yes, how is this done? 2. Which actors do you work with in the community? 3. What are their roles? 4. Any community support mechanisms/initiatives? 5. Challenges at community level 6. What is your comment | | |
| 3.6 | What referral mechanisms are in place?   1. When unable to treat 2. How effective are these mechanisms? | | |
| 3.7 | What challenges do you face in providing the best care for LF patients? (Probe for)   1. Was not aware I needed to provide this service 2. Lack of medication/supplies 3. Lack of training 4. Lack of human resources 5. Poor supervision or support 6. Poor motivation 7. Too many patients 8. Patients don’t present to facility 9. Never encountered a person with lymphedema 10. Other; specify: ____________________________________ | | |
| 3.8 | What recommendations do you have for improving access to LF morbidity services? (Probe for)   1. Improve supervisory support and communication 2. Increase human resources 3. Increase staff motivation 4. Improve training for personnel 5. Increase in number of personnel trained 6. Provide more supplies for patients; specify: ___________________ 7. Implement outreach program 8. Decrease cost of treatment 9. Increase awareness of program 10. Engage community 11. Other; specify: ___________________ | | |
| 3.9 | What recommendations do you have for integrating LF morbidity services into other diseases that you treat in this facility? PROBE ON:   1. Community level recommendations 2. Facility level recommendations 3. Provincial level recommendations 4. National level recommendations | | |

**HEALTH PROVIDERS/COMMUNITY HEALTH WORKERS IDI GUIDE.**

Date: Time:

Interviewer: Health provider ID:

Health Facility/District:

Sex: Age:

Education level: Length of stay in the area:

Position: Number of years practicing:

| **No.** | **Question** |
| --- | --- |
| **SECTION 1: SERVICE UTILIZATION** | |
| 1.1 | How would describe LF management services at the facility/in this district? |
| 1.2 | What challenges do you face in improving access to MMDP services for LF patients? (Probe on)   1. Capacity building of providers 2. Supplies and equipment 3. Lack of sufficient staff 4. Patient and family attitudes 5. Staff motivation |
| 1.3 | In your opinion, why don’t patients seek services at the facility/in this district? (Probe on)   1. Stigma 2. Mobility 3. Transport 4. Paying for services |
| 1.4 | What recommendations do you have for improving access to LF morbidity services? (Probe on)   1. Community level recommendations; community health workers 2. Facility level recommendations 3. Provincial level recommendations 4. National level recommendations |
| 1.5 | What recommendations do you have for integrating LF morbidity services into other diseases that you treat in this facility/in this district? (Probe on)   - 1. Community level recommendations; community health workers   2. Facility level recommendations   3. Provincial level recommendations   4. National level recommendations |

**HEALTH PROVIDERS/COMMUNITY HEALTH WORKERS IDI GUIDE.**

Date: Time:

Interviewer: Leader ID:

Village: Health Facility/District:

Sex: Age:

Education level: Length of stay in the area:

| **No.** | **Question** | **Code** | **Response** |
| --- | --- | --- | --- |
| **SECTION 1: SERVICE UTILIZATION** | | | |
| 1.1 | What are the perceptions within your community of the ways in whi people get the LF? | | |
| 1.2 | What are community perceptions around hydrocele/ lymphoedema which are associated with LF? | | |
| 1.3 | What LF morbidity management services are available? | | |
| 1.4 | How would describe accessibility to these services? | | |
| 1.5 | What factors influence access to services? Probe for stigma etc | | |
| 1.6 | How can we reduce stigma around morbidity associated with LF in your community? | | |
| 1.7 | How describe social support structures for patients in your community? | | |
| 1.8 | How can we increase social support for patients in your community? | | |
| 1.9 | What recommendations do you have for improving access to LF morbidity services for people affected in your community? | | |
