# Supplementary material for: Health beliefs and health seeking behavior towards lymphatic filariasis morbidity management and disability prevention services in Luangwa District, Zambia: Community and provider perspectives
Source: PLoS Negl Trop Dis. 2021 Feb 22;15(2):e0009075. doi: 10.1371/journal.pntd.0009075 (PMC7932505; doi:10.1371/journal.pntd.0009075)
Supplement: Attachment — Submitted filename: PNTD-D-20-00131_reviewer (1).pdf [file pntd.0009075.s002.pdf]

PLOS Neglected Tropical Diseases

Access to morbidity management and disability prevention services for lymphatic filariasis in Luangwa district, Zambia: A mixed methods study.

--Manuscript Draft--

**Manuscript Number:**	PNTD-D-20-00131
**Full Title:**	Access to morbidity management and disability prevention services for lymphatic filariasis in Luangwa district, Zambia: A mixed methods study.
**Short Title:**	Morbidity management & disability prevention services for lymphatic filariasis.
**Article Type:**	Research Article
**Keywords:**	Implementation Research; Lymphatic Filariasis; Morbidity management and disability prevention
**Abstract:**	Introduction<br>Morbidity management and disability prevention (MMDP) services are essential for the management of lymphoedema, elephantiasis and hydrocele that result from lymphatic filariasis (LF) infection. However, there is little information on the access and utilization of MMDP services in Zambia. This study sought to evaluate in status of LF morbidity, utilization and factors affecting access to MMDP services in Luangwa District, Zambia.<br>Methods<br>A concurrent mixed methods study that included a descriptive cross-sectional survey of 237 patients, 4 Focus group discussions and 20 interviews were conducted. Data that was collected included knowledge of LF morbidity, existing morbidity management practices, health seeking behavior, factors affecting access to MMDP services and potential areas of integration of MMDP services into the primary health care system.<br>Findings<br>The most common LF morbidity in the district was hydrocele. There were low levels of knowledge on the causes, symptoms and disease management practices among the different stakeholders. It was common for patients to seek care from both health facilities and traditional healers. 51.0% of hydrocele patients had sought care from a medical facility and 62.5% had gone traditional healers and utilization of surgeries for hydrocele was poor. The main challenges to access included, limited understanding of the disease, lack of awareness of existing MMDP services, unfavorable cultural and social norms, poor motivation to maintain disease management practices and inadequate resources at health facilities. Opportunities for integration included embedding aspects of MMDP services in community structures, existing health services and building capacity both health providers and community health workers.<br>Conclusion<br>Improving access to morbidity management and disability prevention services for LF will require national programmes to effectively map patients and services within health systems. This will streamline existing services and create the much-needed demand for vulnerable and disadvantaged populations.
**Additional Information:**	
**Question**	**Response**
**Financial Disclosure**<br><br>Enter a financial disclosure statement that describes the sources of funding for the work included in this submission. Review the submission guidelines for detailed requirements. View published research articles from *PLOS NTDs* for specific examples.<br><br>This statement is required for submission	This study was funded by the Coalition for Operational Research on Neglected Tropical Diseases (COR-NTD),(https://www.ntdsupport.org/cor-ntd/ntd-connector/term/ntdsc), under Grant Number:NTD-SC #163D and was awarded to CM. The funders had no role in study design, data collection and analysis, decision to publish, or preparation of the manuscript.

Powered by Editorial Manager® and ProduXion Manager® from Aries Systems Corporation

and **will appear in the published article** if the submission is accepted. Please make sure it is accurate.<br><br>**Unfunded studies**<br><br>Enter: *The author(s) received no specific funding for this work.*<br><br>**Funded studies**<br><br>Enter a statement with the following details:<br>• Initials of the authors who received each award<br>• Grant numbers awarded to each author<br>• The full name of each funder<br>• URL of each funder website<br>• Did the sponsors or funders play any role in the study design, data collection and analysis, decision to publish, or preparation of the manuscript?<br>• **NO** - Include this sentence at the end of your statement: *The funders had no role in study design, data collection and analysis, decision to publish, or preparation of the manuscript.*<br>• **YES** - Specify the role(s) played.<br><br>* typeset	
**Competing Interests**<br><br>Use the instructions below to enter a competing interest statement for this submission. On behalf of all authors, disclose any competing interests that could be perceived to bias this work—acknowledging all financial support and any other relevant financial or non-financial competing interests.<br><br>This statement **will appear in the published article** if the submission is accepted. Please make sure it is	The authors have declared that no competing interests exist.

accurate. View published research articles from *PLOS NTDs* for specific examples.<br><br>**NO authors have competing interests**<br><br>Enter: *The authors have declared that no competing interests exist.*<br><br>**Authors with competing interests**<br><br>Enter competing interest details beginning with this statement:<br><br>*I have read the journal's policy and the authors of this manuscript have the following competing interests: [insert competing interests here]*<br><br>* typeset	
**Data Availability**<br><br>Authors are required to make all data underlying the findings described fully available, without restriction, and from the time of publication. PLOS allows rare exceptions to address legal and ethical concerns. See the PLOS Data Policy and FAQ for detailed information.<br><br>A Data Availability Statement describing where the data can be found is required at submission. Your answers to this question constitute the Data Availability Statement and **will be published in the article**, if accepted.	Yes - all data are fully available without restriction

**Important:** Stating 'data available on request from the author' is not sufficient. If your data are only available upon request, select 'No' for the first question and explain your exceptional situation in the text box.<br><br>Do the authors confirm that all data underlying the findings described in their manuscript are fully available without restriction?	
**Describe where the data may be found in full sentences. If you are copying our sample text, replace any instances of XXX with the appropriate details.**<br><br>• If the data are **held or will be held in a public repository**, include URLs, accession numbers or DOIs. If this information will only be available after acceptance, indicate this by ticking the box below. For example: *All XXX files are available from the XXX database (accession number(s) XXX, XXX.*).<br>• If the data are all contained **within the manuscript and/or Supporting Information files**, enter the following: *All relevant data are within the manuscript and its Supporting Information files.*<br>• If neither of these applies but you are able to provide **details of access elsewhere**, with or without limitations, please do so. For example:<br><br>*Data cannot be shared publicly because of [XXX]. Data are available from the XXX Institutional Data Access / Ethics Committee (contact via XXX) for researchers who meet the criteria for access to confidential data.*<br><br>*The data underlying the results*	The data underlying the results presented in the study are available from Taskforce for Global Health and will be made available through https://www.ntdsupport.org/cor-ntd/ntd-connector/term/ntdsc at the completion of all study activities.

presented in the study are available from (include the name of the third party and contact information or URL).<br>• This text is appropriate if the data are owned by a third party and authors do not have permission to share the data.<br><br>* typeset	
Additional data availability information:	

Manuscript

Click here to download Manuscript Acess to MMDP Services in Luangwa .docx

Full title: Access to morbidity management and disability prevention services for lymphatic filariasis in Luangwa district, Zambia: A mixed methods study.

Short title: Morbidity management & disability prevention services for lymphatic filariasis

Authors and affiliations

Patricia Maritim[1*], Adam Silumbwe[2¶], Joseph Mumba Zulu[1¶], George Sichone[3¶], Charles Michelo[6¶]

[1] Department of Health Promotion and Education, University of Zambia School of Public Health, Lusaka, Zambia.

[2] Department of Health Policy and Management, University of Zambia School of Public Health, Lusaka, Zambia.

[3] Participatory Research and Innovations Management, Lusaka, Zambia.

Corresponding Author

[*] Patricia Maritim: triciamarie20@gmail.com

[¶] These authors contributed equally to this work.

[&] These authors also contributed equally to this work

Abstract

Introduction

Morbidity management and disability prevention (MMDP) services are essential for the management of lymphoedema, elephantiasis and hydrocele that result from lymphatic filariasis (LF) infection. However, there is little information on the access and utilization of MMDP services in Zambia. This study sought to evaluate in status of LF morbidity, utilization and factors affecting access to MMDP services in Luangwa District, Zambia.

Methods

A concurrent mixed methods study that included a descriptive cross-sectional survey of 237 patients, 4 Focus group discussions and 20 interviews were conducted. Data that was collected included knowledge of LF morbidity, existing morbidity management practices, health seeking behavior, factors affecting access to MMDP services and potential areas of integration of MMDP services into the primary health care system.

Findings

The most common LF morbidity in the district was hydrocele. There were low levels of knowledge on the causes, symptoms and disease management practices among the different stakeholders. It was common for patients to seek care from both health facilities and traditional healers. 51.0% of hydrocele patients had sought care from a medical facility and 62.5% had gone traditional healers and utilization of surgeries for hydrocele was poor. The main challenges to access included, limited understanding of the disease, lack of awareness of existing MMDP services, unfavorable cultural and social norms, poor motivation to maintain disease management practices and inadequate resources at health facilities. Opportunities for integration included embedding aspects of MMDP services in community structures, existing health services and building capacity both health providers and community health workers.

Conclusion

Improving access to morbidity management and disability prevention services for LF will require national programmes to effectively map patients and services within health systems. This will streamline existing services and create the much-needed demand for vulnerable and disadvantaged populations.

Author Summary

Lymphatic filariasis (LF) infection if untreated usually results in fluid accumulation in the limbs or breasts (lymphoedema) or genitalia (hydrocele) that is painful and causes great discomfort. Morbidity management and disability prevention (MMDP) strategies such as surgery for hydrocele, treatment of acute attacks and management of lymphoedema are necessary for the management of the advances stages of LF. However very few countries including Zambia have adequate information on the availability of MMDP services and quality of care for LF patients. This study explored the utilization of MMDP services and factors that influence access to appropriate care in a highly endemic region, Luangwa District, Zambia between February and April 2019. Utilization of MMDP services was low. Factors affecting access to MMDP services included lack of awareness of existing services, misconceptions about the disease, social and cultural norms, gender, availability of resources and adherence to disease management practices. Potential avenues for the integration of MMDP services into the existing health system include included embedding aspects of MMDP services in community structures, existing health services and building capacity both health providers and community health workers.

Background

Lymphatic filariasis (LF), a neglected tropical disease, causes permanent disability through chronic manifestations of lymphoedema and hydrocele accounting for 1.36 million disability adjusted life years [1]. Globally more than 890 million people living in 49 endemic countries face the risk of infection and an estimated 40 million have chronic manifestations of the disease [2]. Diseases control programmes have mostly targeted the interruption of LF transmission through mass drug administration (MDA) whilst placing less emphasis on promoting morbidity management and disability prevention services (MMDP) for those presenting with chronic manifestations. The basic care package for MMDP services includes individual treatment to destroy microfilaria, treatment for episodes of adenolymphangitis (acute attacks), management of lymphoedema to prevent disease progression and surgery for hydrocele [3]. Since 2000, MDA for LF programs have delivered 7.7 billion cumulative number of treatments to people living in endemic areas [2], whilst fewer lymphoedema and hydrocele patients have accessed to MMDP services in the same period.

According to the World Health Organization (WHO), MMDP services should be included in the basic primary healthcare package, and constitute an indicator for equity in progress towards SDG 3.8 and universal health coverage. However, progress towards establishing and streamlining MMDP services still remains considerably slow, particularly in the Africa, which accounts for a considerable proportion of the LF burden. Out of the 34 LF endemic countries in the African region, only 12 have indicated as having MMDP services for LF patients. WHO reports that even data on MMDP services is rarely captured across countries. Global efforts to eliminate LF through MDA are likely to stop beyond 2020, and strategic direction of diseases control will most likely pivot towards MMDP services.

In Zambia, LF is a public health concern as 87 of 118 districts are considered endemic [2, 4]. MDA for LF was first piloted in Western Province in 2014, and then scaled up nationally in 2015 with annual campaigns to be implemented until 2019. In 2018, national MDA for LF coverage was reported at 90.8 % [2]. Morbidity mapping of LF patients has been concurrently conducted with the implementation of MDA for LF campaigns. The mapping exercise has shown that there are many cases of hydrocele and lymphedema spread across all ten provinces in the country. However, there is limited information of what MMDP services are available, where and how LF patients are accessing them.

The Elimination of Neglected Tropical Diseases National Masterplan 2019-2023 has placed huge emphasis on disability prevention due to LF. However, Zambia currently lacks a comprehensive national MMDP strategy for LF or suitable indicators to monitor the provision of MMDP services [4]. As part of efforts to generate evidence to guide MMDP strategy formulation, the Ministry of Health in collaboration with the University of Zambia, School of Public Health developed a pilot initiative using implementation research approaches to identify the most appropriate mechanisms through which MMDP services can be streamlined and integrated into existing primary health care systems in Luangwa District to inform potential scale up.

Implementation research plays a vital role in Neglected tropical disease control programmes as it makes it possible to identify optimum conditions that promote implementation success. By exploring the process through which disease control interventions are implemented and the ways in which contextual factors may affect implementation. The pilot initiative used an embedded implementation research approach whereby a range of stakeholders including programme implementers, researchers and communities living in the endemic district were involved in all phases of the pilot initiative from problem identification, mapping of the status of MMDP services, validation of findings, intervention development and implementation.

This study presents the findings of a preliminary baseline assessment conducted prior to the development and implementation of the pilot initiative. Specifically, this paper reports on the perspectives of health providers, community members and patients on their knowledge of LF morbidity, morbidity management practices, health seeking behavior, factors affecting access to MMDP services and potential areas of integration of MMDP services into the existing primary health systems in Luangwa District. This information would be used to tailor the components of the MMDP intervention such as the design training curriculums, health education and community sensitization materials, strengthening the data management and referral structures as well as identification of potential areas for integration.

Methods

Study design

This was a concurrent mixed method study conducted as a formative assesment for a larger study that sought to develop and implement an integrated health system intervention to improve access of MMDP services for LF patients within Luangwa District of Lusaka, Zambia.

Study setting

The study was conducted between February and April 2019 in Luangwa District, which has one of the highest prevalence of the Circulating Filarial Antigen [5] as it found along the Luangwa river valley. The district is predominantly rural with an estimated population of 31,665, covered by a total of 17 health facilities.

Baseline mapping of patients

Prior to conducting the study, a census of LF was done to map the distribution of cases across the health facility catchment areas and formed the basis for sampling for the data collection. Community health workers (CHWs) went from household to household identifying patients who exhibited signs of lymphoedema, elephantiasis and hydrocele.

Patient descriptive cross-sectional survey

A total of 237 patients were identified during the mapping exercise all of whom were invited to take part in the study. The recruitment of study participants for the patient survey was done by the CHWs, within the health facilities and surrounding communities with the help of the study team and staff from the District Health Office. Trained CHWs administered a structured KAP questionnaire. All patients who were identified during the mapping exercise agreed to take part in the study. Interviews were held within the homes LF patients to ensure that their privacy and confidentiality was upheld.

Focus group discussions

There were 4 focus group discussions (FGD) held in Luangwa Boma and Mphuka health facility catchment areas that had the highest number of patients in the district. The FGDs were an opportunity to collect information on community level understanding of the chronic stages of LF, practices used to manage the conditions and access to health care.

Participants were conveniently sampled from the communities living in the catchment areas and included LF patients. Potential study participants were identified by community health workers working in the catchment areas and were invited by telephone to participate by the health facility in-charges. In terms of composition, the FGDs were mixed with regard to age but were separated by gender (2 male and 2 female.) The gender separation was essential given that in the communities living in the area, hydrocele is considered a sensitive topic of discussion and this would encourage community members to speak freely. Each FGD had between 10 and 12 participants. The FGDs were conducted at the health facility but away from patient areas to ensure the privacy of the participants and avoid interruptions. The FGDs lasted for 1 hour 30 minutes to 2 hours.

Interviews

Interviews were conducted with district neglected tropical disease focal point persons (n=2) as well as community health workers (n=8), health facility staff (n=8) and traditional leaders (n=8) from 8 health facility catchment areas which had the highest number of LF patients in the district - Luangwa Boma, Mpukha, Katondwe, Kanemela, Chitope, Kasinsa, Luangwa District Hospital and Mandombe. Participants were purposively sampled based on their involvement in the implementation of LF elimination programmes in the area. The interviews were used identify health system factors related to access of MMDP services in the district. A total of twenty interviews were done with participants invited to take part either through telephone calls or face to face. Interviews were conducted at the respective health facilities and the Luangwa district Health Offices. Interviews lasted between 45 minutes to an hour.

Data collection

The KAP questionnaire included questions on knowledge of lymphoedema such as symptoms, causes and prevention measures, morbidity management practices, health seeking behaviour and factors affecting their access to care. Factors affecting access to care in the questionnaire, Interview and FGD guides were adapted from Levesque et al's conceptual framework on patient centered access to health care [6]. Prior to data collection the data collection tools were pretested to ensure their suitability and changes made where necessary. All FGDs and interviews were audio recorded with the consent of the study participants. In addition, field notes were taken during the course of the interviews. Data collection was done in English and Nyanja to ensure that all participants were able to articulate their perspectives as comprehensively as possible.

Data analysis

All recordings were transcribed verbatim and those done in Nyanja were translated to English. A framework analysis approach was applied to analyze the data [7] using Levesque et al's conceptual framework on patient centered access to health care and has been used in health

systems and policy research [6, 8, 9]. The framework defines access as the “opportunity to reach and obtain appropriate health care services in situations of perceived need for care.” It proposes that from a health care provider perspective, access to care can be categorized into five dimensions; approachability, acceptability, availability and accommodation, affordability and appropriateness. Barriers and facilitators can impact these dimensions which in turn affects an individual’s ability to utilize health services. By affecting their ability to perceive, ability to seek, ability to reach, ability to pay and ability to engage with the available health services [6]. These demand and supply side dimensions formed the coding tree from which predetermined themes and sub themes were extracted using both inductive and deductive methods to ensure that existing and emergent themes were exhaustively identified. Preliminary coding of the transcripts was done by PM and AS. JZ reviewed the coding lists to ensure that identified codes were aligned to the to the framework. Subsequently PM, AS and JZ jointly reviewed the codes and developed the final codebook.

Quantitative data from the survey was entered in excel template, cleaned to promote data correctness and thereafter exported to STATA version 13 where it was analysed. Baseline measures of the different knowledge, awareness and practice questions were summarised using descriptive statistics. The data was reported as proportions and frequencies. The data the qualitative and quantitative analysis was then integrated to provide a comprehensive picture of MMDP in the district. Comparisons to other studies done in LF endemic countries looking at MMDP, was done to examine the congruence of the identified factors with existing literature and to gauge the strength of the analysis.

Validation of study findings

A stakeholder meeting was held to validate the findings of the study and to confirm that the information that they provided was accurately reflected in the transcripts. Fifty participants took

part in the workshop including Local Chiefs, traditional leaders, Ministry of health staff and CHWs. Another outcome of the stakeholder validation meeting was the identification of avenues for potential integration of MMDP services into the health system within the district. Participants worked together to identify health system strengthening approaches through identifying underlying mechanisms necessary for successful integration by detailing key health messages, necessary actors, existing referrals pathways, reporting systems, monitoring and evaluation systems and mentorship for health workers.

Ethics statement

Ethical approval was sought from University of Zambia Biomedical Research Ethics Committee (REF.017-11-18) and the National Health Research Authority under the Ministry of Health, Zambia. All participants were informed of the purpose of the assessment, details of study procedures including freedom to withdraw, potential benefits and risks, prior to the commencement of data collection. Thereafter written informed consent was obtained as all participants were 18 years and above.

Results

Characteristics of patients.

There were 237 individuals sampled during the mapping exercise and the patient survey, and from these, 199 (85.4%) had hydrocele, 27 (11.6%) had lymphedema and 7 (3.0%) had both lymphoedema and hydrocele. A majority of the participants were male (90.7%). The mean age was 46.9 (S.D 21.4). Most of the survey participants practiced Christianity (96.2%), were married (66.2%) and had lived in the area for more than 5 years (86 %.) For most of the study participants the highest level of education that they had attended was primary school (70.5%) as shown in Table 1.

Table 1. Demographic characteristics of LF Patients in Luangwa District

Variable	Proportion<br><br>(total sample=237)
**Gender**	
1= Female<br><br>2= Male	9.3% (22)<br><br>90.7% (215)
**Education**	
1= No education<br><br>2= Primary School<br><br>3= Junior High school<br><br>4= Secondary school<br><br>5= Vocational training<br><br>6= University<br><br>7= Other	14.8% (35)<br><br>70.5% (167)<br><br>3.4% (8)<br><br>10.1% (24)<br><br>0.4% (1)<br><br>0.4% (1)<br><br>0.4% (1)
**Marital Status**	
1= Single<br><br>2= Married<br><br>3= Divorced<br><br>4= Widowed	22.8% (54)<br><br>66.2% (157)<br><br>3.4% (8)<br><br>7.6% (18)
**Income Source**	
1= Unemployed<br><br>2= Agricultural activities<br><br>3= Day worker<br><br>4= Small scale enterprise<br><br>5= Private sector<br><br>6= Civil Servant<br><br>7= Other (charcoal, carpentry)	30.8% (73)<br><br>52.3% (124)<br><br>2.5% (6)<br><br>0.4% (1)<br><br>2.1% (4)<br><br>0.4% (1)<br><br>11.4% (27)

**Monthly Income**	
1= <1000 kwacha<br><br>2= 1000-4000 kwacha<br><br>3= 4000-10000 kwacha<br><br>4= > 10000 kwacha<br><br>5= I don't know	68.8% (161)<br><br>4.7% (11)<br><br>0.4% (1)<br><br>3.8% (9)<br><br>22.2% (52)
**Duration they have lived in study area**	
<1 year<br><br>1-5 years<br><br>> 5 years<br><br>5*	4.2% (10)<br><br>8.4% (20)<br><br>86% (203)<br><br>1.3% (3)
**Available transport**	
1= Car<br><br>2= Bicycle<br><br>3= Motorbike<br><br>4= Walking<br><br>5= Other	3 (1.3)<br><br>46 (19.8)<br><br>1 (0.4)<br><br>176 (75.9)<br><br>6 (2.6)
**Transport to the health facility**	
1= Car<br><br>2= Bicycle<br><br>3= Motorbike<br><br>4= Walking<br><br>5= Other	2 (0.9)<br><br>12 (5.4)<br><br>19 (8.6)<br><br>62 (28.1)<br><br>126 (57.0)

Knowledge of lymphoedema, hydrocele and elephantiasis.

The communities have local names for hydrocele, which is referred to as *tumbu*. There were various cultural beliefs surrounding the causes of the chronic stages of LF. During the FGDs, some of the common causes of lymphoedema and elephantiasis that were described included the belief that the conditions were hereditary and was passed through family lines, eating food that had not been warmed properly, contact with animal faeces, using traditional medicine such as local herbal aphrodisiacs *mutoto* and children vomiting on their mothers during breastfeeding. Reported causes of hydrocele were largely similar to those given for lymphoedema but also included having sexual contact with women who were menstruating or who've had a miscarriage, being bewitched, men using pounding sticks after they have been used by women, sitting on chairs which patients have used and sitting on stones that women use during food preparation.

> "…. Mostly what brings this disease is the careless sitting on things. For example, sitting on the stones that women use for fire and also sitting on the stone that women use to grind or crush mealie meal……" [FGD1_ Community Members].

Some of the FGD participants were able to correctly identify mosquitoes, which are common in the area because of the presence of River Luangwa, as the disease-causing vectors. This could be due to community sensitisation exercises done as part of mass drug distribution rounds conducted between 2015 and 2018. However only 11.4% patients could correctly identify mosquitoes as the vector that spreads the disease. When asked to rate their general understanding of their conditions, a majority of the patients stated that they had a poor understanding; (86.7% of lymphoedema and 77% of hydrocele patients). Most of the patients and participants of the FGDs reported knowing patients who lived in their villages or were members of their families and had lymphedema, elephantiasis or hydrocele.

Knowledge levels among traditional leaders, community-based volunteers and health care providers interviewed differed greatly. Generally, those who had been heavily involved in LF

disease control activities such as MDA rounds were more likely to be knowledgeable about the causes and symptoms of the disease, because they had received training and information to guide implementation. Most of the traditional leaders interviewed reiterated the prevailing community beliefs reported during the FGDs. For instance, during the stakeholder validation meeting, a chief who was in attendance sought clarification on the means through which hydrocele is transmitted and whether it be cured.

> "……. Here we believe that if you are passing where there are faeces of animals especially this rain season whether bush animals or village animals and it can be goats, cattle, sheep, pigs, elephants or other animals the disease goes inside the nails making your legs swollen so even if you treat hydrocele it can't be healed and that person will die at that old age with swollen legs like that…." [KII5_ Traditional Leader].

Despite some healthcare providers having a basic understanding of the conditions and the correct means of providing care, a considerable proportion of those who were interviewed and who took part in the stakeholder validation meetings, acknowledged that they limited knowledge of the disease and its management. Healthcare providers who came into regular contact with patients coming to the facility or through community case identification exercises were better at identifying signs and symptoms of lymphoedema, elephantiasis and hydrocele.

MMDP Practices and utilization of MMDP services

Health facility MMDP services. At the health facilities, available services are general in nature without specific stand-alone activities for hydrocele and lymphedema. Not all health facilities in the area have adequate resources to conduct hydrocelectomies and referrals are usually made to either Katondwe Mission Hospital or Luangwa District Hospital. Severe cases are referred to the University teaching Hospital in Lusaka. In the case of lymphoedema, the most readily available services are pain relief and general health education with IEC materials on display in busy

outpatient areas. The healthcare providers expressed that they also provided antibiotics to prevent secondary infection as a result of acute attacks as well as lymphatic draining to reduce fluid density. Annual mass drug administration rounds were done to interrupt disease transmission. Despite surgical interventions being available at health facilities, utilisation was low.

In terms of utilization of available services, 51,1% of LF patients had sought care for their condition at the health facility before. It was common to find that patients would go to the health facility and to traditional healers. Community members pointed out that patients were more likely to go to the traditional healers because they felt as though their remedies were a more permanent solution compared to when they went to the facilities. Since once the medication prescribed at the facility ran out their symptoms would reappear. One of the traditional healers who took part in the validation meeting and had hydrocele himself, pointed out how common it was for LF patients to seek remedies from him. Slightly more than half of the lymphoedema patients (53.1%) had previously sought medical help for their condition and 71.9% who had gone to a traditional healer. Similarly, 51.0% of hydrocele patients had sought care from a medical facility and 62.5% had gone traditional healers. However, the tattoos and herbs administered by the traditional healers would exacerbate the acute attacks forcing the patients to go to the health facility.

The number of patients who had had surgery to reduce the swelling due to hydrocele was quite low at 14.3%. However, 58.6% of them were taking doxycycline as an alternative to surgery. Even though the service is provided for free once they get a referral, the main reasons provided for failure to have the surgeries done were insufficient money (59.2%) and unaware of services (14.1%).

Home based care strategies. The most common home-based strategies that health care facility staff advised lymphoedema patients to do were exercise, elevation, improved hygiene owing to the low economic situations that most of the patients came from, wearing baggy clothes, wound management to prevent secondary infections and pain killers just to help the patients deal with the immediate pain. Nevertheless, patients' knowledge of home-based care strategies that could prevent their lymphoedema from worsening from the patient survey was found to be low. A majority of lymphoedema patients (73.5%) had never received any information on how to manage their condition, despite more than half of them having been visited by a community-based volunteer. With regards to availability of supplies to be able to effectively manage their conditions from home, of the 22 patients with leg lymphedema only one had appropriate footwear at home. Furthermore only 15.6% had bandages and 29% had either soap or water for washing wounds. None of them had antibiotic ointments. Moreover 60% of the patients admitted to not practicing any home-based care for their condition. Whereas those who did mostly did exercises or used soap and water to clean affected areas. Community member knowledge of home-based care strategies for lymphoedema and acute attacks was also found to be low.

Factors affecting access to MMDP services

Approachability and ability to perceive need for care

Lack of awareness of existing services. A recurring theme across the focus group discussions and the interviews is that most community members are not aware that there were MMDP services available at the health facilities or that they could be able to take preventive measures through home-based care strategies. Health talks provided by community health workers with the help of LF patients as champions are being used as an opportunity to disseminate this information. Resulting in patients who were initially hesitant about going to the health facility. Health

education provided during outreach activities that were conducted as part of MDA were an opportunity through which healthcare providers provided information on the diseases and more community member were open to having their households sprayed during Indoor Residual Spraying (IRS) exercises as part of prevention measures. Outside of MDA, community sensitisation exercises tend to focus on diseases such as malaria, HIV/AIDS and maternal, newborn and child related conditions as LF is not perceived to be a public health concern in the district.

Belief about the disease. Due to the communal belief that LF is hereditary with some patients having seen their parents and grandparents exhibiting symptoms and not seeking care, some patients felt that it was unnecessary to seek care. Additionally, some patients were afraid to go the health facility because they felt that their conditions had progressed to the more advanced stages and that the services they would receive would not alleviate their symptoms

Misconceptions about the treatment process. In the case of hydrocele some thought that the surgery provided would leave them in wheelchairs making it difficult for them to undertake their regular duties.

Acceptability and ability to seek health care

Cultural beliefs. Hydrocele and lymphoedema are normalised in the district. Though LF patients were considered to be in a pitiful state, FGD participants pointed out that it is common to see hydrocele patients taking part in businesses such as fish mongering and farming.

Social norms. Having hydrocele is viewed as a marker of high social standing and men who had it are more likely to be chosen as headmen as they are perceived to be old and wise. This has acted as a deterrent to patients seeking care. A key deterrent to the uptake of surgical interventions among hydrocele patients was the fear of loss of respect by their spouses. Some of the patients thought that once the procedures had been done they would be incapable of having

children which would make their wives not take them seriously. Some women in the communities whose husbands had hydrocele had left them due to lack of sexual satisfaction.

> "…. When they get sick they don't even go to the hospital because they are respected so much. Yes, they respect him a lot even if you reach somewhere he will be the first one to be given the chair just because of what they are seeing, respecting him a lot that one who doesn't have, so mostly if you become a headman they believe you are supposed to have hydrocele and you should become the head of the family you are supposed to have that so that even if you this is our leader….."[KII2_ Traditional Leader].

Gender. According to the health care providers, it is more difficult to get men to come to the health facility, as compared to women and children despite hydrocele being the most common LF manifestation in the district. During the focus group discussions community members, shared the view that certain diseases, particularly to do with private parts are not meant to be shown to the opposite sex. As such hydrocele patients are embarrassed to access services and even if they do, they do not easily open up especially if they are being attended to by female health care providers. One of the suggestions given by community members on ways of increasing patients ability to seek care was having male healthcare providers handle hydrocele patients.

Stigmatisation. Fear of stigmatisation also inhibits patients' ability to seek care. During regular case identification exercises by healthcare providers, known hydrocele patients, who are approached for referral to the health facility would either deny having the condition or request to talk to them in private because they are afraid that they would be laughed at if other community members found out that they had hydrocele. The poor health seeking habits are compounded by low levels of education among communities living in the district.

Availability, accommodation and ability to reach health care

Geographic location. Luangwa District is very remote and a portion of the district is covered by the Luangwa National Park. As a result, some communities in the district have to travel long distances of up to 20 kilometers and more to the nearest health facility and their access may be inhibited by wildlife attacks such as elephants from the Luangwa National Park. In addition, roads to the health facilities are sometimes impassable and the most common means of transport is bicycles which are inappropriate to transport lymphoedema and hydrocele patients. As such some patients choose to stay home rather than go to the health facility.

Mobility. In addition, the district borders Mozambique and some patients are involved in economic activities such as trade requiring them to travel. Therefore, when healthcare providers conduct outreach and follow up visits at community level to provide some MMDP services they do not find them at their homes.

Adequate resources. Procedures like hydrocelectomies require more qualified personnel like a medical doctor, who were not common in remote areas where these patients are found. A majority of the healthcare providers interviewed had not received any special training on how to handle and manage patients with hydrocele and lymphedema, except for the normal training provided during their formal training. They felt that they needed additional training to effectively provide quality care to patients. In addition, medical supplies such as diagnostic tests necessary for lymphedema and hydrocele management in most of the health facilities was inadequate.

Affordability and ability to pay for health care

Direct costs. Though patients are not expected to pay for the care that they receive at the health facility, there is a perception among patients that they are still required to pay for interventions such as the hydrocele surgery which dissuades them from seeking care.

Indirect costs. In the event that during assessment a patient's case is determined to be complicated, they are referred to University Teaching Hospital in Lusaka. For most patients the

associated costs needed for them to obtain the care they need including hiring transportation and out of pocket hospital expenses act as barriers to accessing care. The health facility catchment areas also cover neighboring villages in Mozambique and the residents in these villages usually save over time to be able to access care.

> "…. If maybe the family doesn't have a bicycle and they have lymphoedema, some they have difficulties in walking so it is difficult for them to come and access the services if its not near and especially those from across the border they have to like save up money before they can come this side…." [IDI1_ Healthcare provider].

Opportunity costs. Moreover, most of those who live in the district are poor and when deciding how best to prioritize available resources, they opt to use the minimal resources that are at their disposal for food and basic necessities first before considering spending on health care. For most of those involved in income generating activities such as fishing and trade, the opportunity cost associated with taking time off to go to a health facility is not a preferred choice. According to community members, the prevalent use of traditional healers and witchdoctors, often results in sores and secondary infections that force patients to then go to the health facility which increases the resources they spend on looking for effective treatment and management services.

Appropriateness and ability to engage

Adherence to MMDP practices. Progression of the symptoms of hydrocele and lymphoedema are dependent on proper long-term management and care. Patient motivation to maintain home based care practices is critical to ensure that lymphoedema does not progress to elephantiasis and that surgical interventions are undertaken early for hydrocele. A common sentiment expressed during the FGDs and interviews was that many lymphoedema patients lose their motivation to continue going to the facility for MMDP services which they felt was a waste of time, because despite taking up medication over a long period their conditions stayed the same. They did not

view the lack of progression of their symptoms to elephantiasis and fewer acute attacks as favourable treatment outcomes as they wanted their symptoms fully cured.

Integration of MMDP services

Opportunities for integration.

Use of existing community structures. One of the main suggestions provided during the data collection and the stakeholder validation meetings was use of community-based volunteers who are rooted in the community and had been part of training for MDA, to deliver key messages relating to MMDP. The inclusion of traditional leaders and chiefs in engaging community members would help address some of the social and cultural beliefs that inhibited patients from accessing care. Some traditional leaders would act as champions.

Training and capacity building. Provision of training to different health care providers and community-based volunteers, was considered necessary in creating awareness of existing MMDP services at the facilities and different home-based care strategies. Once trained they would be better able to communicate the information to community members during outreach exercise, health talks and when doing follow up visits with patients at their homes. As well as in schools through the School health and Nutrition programme.

Integration into the existing healthcare services. Different suggestions were made by the healthcare providers on which services would be most appropriate for integration. They felt that lymphatic filariasis had some similarities with other diseases. Integrating into malaria programs was proposed due to the similarities in the mode of transmission and ease of tailoring messages around preventive and control measures. Water, Sanitation and Hygiene programmes were also proposed due to the role of proper sanitation and hygiene in the prevention of secondary infection.

DISCUSSION

Lymphatic filariasis infection if untreated results, in advanced stages of lymphoedema, elephantiasis and hydrocele which are not only debilitating but without care may result in permanent disability. Morbidity management and disability prevention services provide an opportunity through which patients could have access to management strategies that ensure their conditions do not progress to more advanced stages. Allowing them to be fully involved in community activities and undertake personal daily tasks.

In this study we explored perspectives towards MMDP practices within Luangwa district across a diverse range of stakeholders from patients to service providers to identify the most appropriate ways of providing care to LF patients within the existing health system in the district using Levesque et al. framework [6]. As well as identify different determinants that may affect the implementation on interventions that are aimed at providing MMDP services. We found that knowledge of the causes of disease, sign/symptoms that mark the different diseases stages and ways in which the disease can be managed through simple home-based care practices among community members was very low. It was still common to find participants in the study who thought that care practices would not have any effect on stopping disease progression. This is despite ongoing health education efforts that are conducted in the area as part of mass drug administration rounds. The negative effect of hydrocele on patients' marriages and sexual lives is similar to what has been found in other settings [10].

Whilst the study was able to highlight the existence of certain healthcare services–like surgery–that LF patients can access at the health facilities, it also demonstrated that access for the patients still remains a huge challenge. The low level of priority given to morbidity management and disability prevention services affects the ability of LF patients to seek appropriate care. Despite the existence of various MMDP guidelines that guide health care providers and support community and home-based care, it is common to find low knowledge levels within endemic

districts. Community factors such as negative traditional beliefs around certain health services like surgery play an important role with regards to shaping access and use of health services. There is a need to provide training opportunities and strengthen referral systems to ensure patients not only get appropriate care but that subsequent follow up is made possible. Furthermore, MMDP programmes should include strategies that seek to empower LF patients by ensuring that they get the required information, to access and use the services at the health facilities.

Acknowledgements

We would like to acknowledge Edson Musonda and Frank Shamilimo from the Ministry of Health who facilitated the study process. This work received financial support from the Coalition for Operational Research on Neglected Tropical Diseases (COR-NTD), which is funded at The Task Force for Global Health primarily by the Bill & Melinda Gates Foundation, by the UK aid from the British government, and by the United States Agency for International Development through its Neglected Tropical Diseases Program.

References

1. Kyu HH, Abate D, Abate KH, Abay SM, Abbafati C, Abbasi N, et al. Global, regional, and national disability-adjusted life-years (DALYs) for 359 diseases and injuries and healthy life expectancy (HALE) for 195 countries and territories, 1990–2017: a systematic analysis for the Global Burden of Disease Study 2017. The Lancet. 2018;392(10159):1859-922.
2. World Health Organisation. Global programme to eliminate lymphatic filariasis: progress report, 2018. . The Weekly Epidemiological Record 11th October 2019. 2019;2019, 94:457-72.
3. World Health Organisation. Lymphatic filariasis: managing morbidity and preventing disability: an aide-mémoire for national programme managers. 2013.
4. Ministry of Health,Zambia. Elimination of Neglected Tropical Diseases National Masterplan 2019-2023. Lusaka, Zambia.: 2019.
5. Mwase ET, Stensgaard A-S, Nsakashalo-Senkwe M, Mubila L, Mwansa J, Songolo P, et al. Mapping the geographical distribution of lymphatic filariasis in Zambia. PLoS neglected tropical diseases. 2014;8(2):e2714.
6. Levesque J-F, Harris MF, Russell G. Patient-centred access to health care: conceptualising access at the interface of health systems and populations. International journal for equity in health. 2013;12(1):18.
7. Ritchie J, Spencer L. Qualitative data analysis for applied policy research. Analyzing qualitative data: Routledge; 2002. p. 187-208.
8. Hailemariam M, Fekadu A, Selamu M, Medhin G, Prince M, Hanlon C. Equitable access to integrated primary mental healthcare for people with severe mental disorders in Ethiopia: a formative study. International journal for equity in health. 2016;15(1):121.

9. Davy C, Harfield S, McArthur A, Munn Z, Brown A. Access to primary health care services for Indigenous peoples: A framework synthesis. International journal for equity in health. 2016;15(1):163.

10. Babu BV, Mishra S, Nayak AN. Marriage, sex, and hydrocele: an ethnographic study on the effect of filarial hydrocele on conjugal life and marriageability from Orissa, India. PLoS neglected tropical diseases. 2009;3(4):e414.
