# Supplementary material for: Health beliefs and health seeking behavior towards lymphatic filariasis morbidity management and disability prevention services in Luangwa District, Zambia: Community and provider perspectives
Source: PLoS Negl Trop Dis. 2021 Feb 22;15(2):e0009075. doi: 10.1371/journal.pntd.0009075 (PMC7932505; doi:10.1371/journal.pntd.0009075)
Supplement: Attachment — Submitted filename: PNTD-D-20-00131_R1 edited.pdf [file pntd.0009075.s004.pdf]

PLOS Neglected Tropical Diseases

Community perspectives towards morbidity management and disability prevention for lymphatic filariasis in Luangwa district, Zambia: A qualitative study.

--Manuscript Draft--

**Manuscript Number:**	PNTD-D-20-00131R1
**Full Title:**	Community perspectives towards morbidity management and disability prevention for lymphatic filariasis in Luangwa district, Zambia: A qualitative study.
**Short Title:**	Morbidity management and disability prevention in Zambia.
**Article Type:**	Research Article
**Keywords:**	Implementation research; Lymphatic Filariasis; Morbidity management and disability prevention, Zambia
**Abstract:**	Background<br>Morbidity management and disability prevention (MMDP) services are essential for the management of elephantiasis, lymphoedema, and hydrocele that result from lymphatic filariasis (LF) infection. However, there is limited information on health beliefs and health seeking behaviour related to MMDP services within LF endemic regions of Zambia. This study sought to document health beliefs and health seeking behaviour for LF MMDP services among communities living in Luangwa District, Zambia.<br>Methods<br>This was an exploratory qualitative study conducted with community members including LF patients, community health workers and healthcare providers. Data was collected through a series of 4 focus group discussions stratified by sex and 26 interviews in-depth interviews. Data were analyzed by thematic analysis using NVivo software.<br>Results<br>The most commonly mentioned causes of the chronic manifestations of LF included; contact with animal faeces, using traditional herbal aphrodisiacs (mutoto) , sexual contact with women who were menstruating or had miscarried and witchcraft. LF patients would opt to visit a traditional healer before going to the health facility. Hydrocele patients were reported to be afraid of hydrocelectomies because they thought they would become infertile or die. Very few of the community members were able to identify any home and facility-based care strategies for LF patients. Health system and cultural barriers to seeking healthcare included; long distances to the health facilities, lack of awareness of existing MMDP services, costs of accessing healthcare services, gender and social norms and fear of stigmatization.<br>Conclusion<br>This study found that health seeking behaviour among LF patients are largely driven by the causes associated with the disease. Ongoing community sensitisation and provider capacity building efforts that seek to create demand for existing morbidity management services at primary health care and household levels, as well as address patients' concerns surrounding access to care are necessary.
**Additional Information:**	
**Question**	**Response**
**Financial Disclosure**<br><br>Enter a financial disclosure statement that describes the sources of funding for the work included in this submission. Review the submission guidelines for detailed requirements. View published research articles from *PLOS NTDs* for specific examples.	This study was funded by the Coalition for Operational Research on Neglected Tropical Diseases (COR-NTD),(https://www.ntdsupport.org/cor-ntd/ntd-connector/term/ntdsc), under Grant Number:NTD-SC #163D and was awarded to CM. The funders had no role in study design, data collection and analysis, decision to publish, or preparation of the manuscript.

This statement is required for submission and **will appear in the published article** if the submission is accepted. Please make sure it is accurate.<br><br>**Unfunded studies**<br><br>Enter: *The author(s) received no specific funding for this work.*<br><br>**Funded studies**<br><br>Enter a statement with the following details:<br>• Initials of the authors who received each award<br>• Grant numbers awarded to each author<br>• The full name of each funder<br>• URL of each funder website<br>• Did the sponsors or funders play any role in the study design, data collection and analysis, decision to publish, or preparation of the manuscript?<br>• **NO** - Include this sentence at the end of your statement: *The funders had no role in study design, data collection and analysis, decision to publish, or preparation of the manuscript.*<br>• **YES** - Specify the role(s) played.<br><br>* typeset	
**Competing Interests**<br><br>Use the instructions below to enter a competing interest statement for this submission. On behalf of all authors, disclose any competing interests that could be perceived to bias this work—acknowledging all financial support and any other relevant financial or non-financial competing interests.<br><br>This statement **will appear in the published article** if the submission is	The authors have declared that no competing interests exist.

accepted. Please make sure it is accurate. View published research articles from *PLOS NTDs* for specific examples.<br><br>**NO authors have competing interests**<br><br>Enter: *The authors have declared that no competing interests exist.*<br><br>**Authors with competing interests**<br><br>Enter competing interest details beginning with this statement:<br><br>*I have read the journal's policy and the authors of this manuscript have the following competing interests: [insert competing interests here]*<br><br>* typeset	
**Data Availability**<br><br>Authors are required to make all data underlying the findings described fully available, without restriction, and from the time of publication. PLOS allows rare exceptions to address legal and ethical concerns. See the PLOS Data Policy and FAQ for detailed information.<br><br>A Data Availability Statement describing where the data can be found is required at submission. Your answers to this question constitute the Data Availability Statement and **will be published in the article**, if accepted.	Yes - all data are fully available without restriction

**Important:** Stating 'data available on request from the author' is not sufficient. If your data are only available upon request, select 'No' for the first question and explain your exceptional situation in the text box.<br><br>Do the authors confirm that all data underlying the findings described in their manuscript are fully available without restriction?	
**Describe where the data may be found in full sentences. If you are copying our sample text, replace any instances of XXX with the appropriate details.**<br><br>• If the data are **held or will be held in a public repository**, include URLs, accession numbers or DOIs. If this information will only be available after acceptance, indicate this by ticking the box below. For example: *All XXX files are available from the XXX database (accession number(s) XXX, XXX.*).<br>• If the data are all contained **within the manuscript and/or Supporting Information files**, enter the following: *All relevant data are within the manuscript and its Supporting Information files.*<br>• If neither of these applies but you are able to provide **details of access elsewhere**, with or without limitations, please do so. For example:<br><br>*Data cannot be shared publicly because of [XXX]. Data are available from the XXX Institutional Data Access / Ethics Committee (contact via XXX) for researchers who meet the criteria for access to confidential data.*	The data underlying the results presented in the study are available from Taskforce for Global Health and will be made available through https://www.ntdsupport.org/cor-ntd/ntd-connector/term/ntdsc at the completion of all study activities.

The data underlying the results presented in the study are available from (include the name of the third party and contact information or URL).<br>• This text is appropriate if the data are owned by a third party and authors do not have permission to share the data.<br><br>* typeset	
Additional data availability information:	

Manuscript

Click here to access/download;Manuscript;Revised Acess to MMDP Services in Luangwa Clean edit.docx

Community perspectives towards morbidity management and disability prevention for lymphatic filariasis in Luangwa district, Zambia: A qualitative study.

Short title: Morbidity management and disability prevention in Zambia.

Authors and affiliations

Patricia Maritim[1*], Adam Silumbwe[2¶], Joseph Mumba Zulu[1¶], George Sichone[3¶], Charles Michelo[4¶]

[1]Department of Health Promotion and Education, School of Public Health, University of Zambia, Lusaka, Zambia.

[2]Department of Health Policy and Management, School of Public Health, University of Zambia, Lusaka, Zambia.

[3]Participatory Research and Innovations Management (PRIM), Lusaka, Zambia.

[4]Department of Epidemiology and Biostatistics, School of Public Health, University of Zambia Lusaka, Zambia.

Corresponding Author

[*] Patricia Maritim: triciamarie20@gmail.com

[¶] These authors contributed equally to this work.

[&] These authors also contributed equally to this work

Abstract

Background

Morbidity management and disability prevention (MMDP) services are essential for the management of elephantiasis, lymphoedema, and hydrocele that result from lymphatic filariasis (LF) infection. However, there is limited information on health beliefs and health seeking behaviour related to MMDP services within LF endemic regions of Zambia. This study sought to document health beliefs and health seeking behaviour for LF MMDP services among communities living in Luangwa District, Zambia.

Methods

This was an exploratory qualitative study conducted with community members including LF patients, community health workers and healthcare providers. Data was collected through a series of 4 focus group discussions stratified by sex and 26 ~~interviews~~ in-depth interviews. Data were analyzed by thematic analysis using NVivo software.

Results

The most commonly mentioned causes of the chronic manifestations of LF included; contact with animal faeces, using traditional herbal aphrodisiacs (*mutoto*), sexual contact with women who were menstruating or had miscarried and witchcraft. LF patients would opt to visit a traditional healer before going to the health facility. Hydrocele patients were reported to be afraid of hydrocelectomies because they thought they would become infertile or die. Very few of the community members were able to identify any home and facility-based care strategies for LF patients. Health system and cultural barriers to seeking healthcare included; long distances to the health facilities, lack of awareness of existing MMDP services, costs of accessing healthcare services, gender and social norms and fear of stigmatization.

Conclusion

This study found that health seeking behaviour among LF patients are largely driven by the causes associated with the disease. Ongoing community sensitisation and provider capacity building efforts that seek to create demand for existing morbidity management services at primary health care and household levels, as well as address patients' concerns surrounding access to care are necessary.

Key words: Lymphatic filariasis, morbidity management, Zambia,

Author Summary

Lymphatic filariasis (LF) infection if untreated results in fluid accumulation in the limbs or breasts (lymphoedema) or genitalia (hydrocele) that is painful and causes great discomfort. Morbidity management and disability prevention (MMDP) strategies such as surgery for hydrocele, treatment of acute attacks and management of lymphoedema are necessary for the management of the advanced stages of LF. However, very few countries including Zambia, have adequate information on the health beliefs and health seeking behaviour of communities living in endemic areas. This study sought to explore community perspectives to morbidity management and disability prevention for LF in a highly endemic region, Luangwa District, Zambia between February and April 2019. Some of the common causes of lymphoedema and hydrocele mentioned were; contact with animal faeces, using traditional herbal aphrodisiacs (*mutoto)*, sexual contact with women who were menstruating or had miscarried and witchcraft. There was limited knowledge of home-based and facility-based care strategies for LF patients. Nevertheless, patients would often go to health facilities after visiting traditional healers and observing no improvement. Barriers to accessing healthcare included; long distances to the health facilities, lack of awareness of existing MMDP services, costs of accessing healthcare services, gender and social norms and fear of stigmatization.

Background

Lymphatic filariasis (LF), a neglected tropical disease, causes permanent disability through chronic manifestations of lymphoedema and hydrocele accounting for 1.36 million disability adjusted life years [1]. Globally more than 890 million people living in 72 endemic countries face the risk of infection and an estimated 40 million have chronic manifestations of the disease [2]. Disease control programmes have mostly targeted the interruption of LF transmission through mass drug administration (MDA) whilst placing less emphasis on promoting morbidity management and disability prevention services (MMDP) for those presenting with chronic manifestations. The basic care package for MMDP services includes individual treatment for episodes of adenolymphangitis (acute attacks), destruction of microfilaria, management of lymphoedema to prevent disease progression, and surgery for hydrocele [3]. Since 2000, MDA programmes for LF programs have delivered 7.7 billion cumulative treatments to people living in endemic areas [2], whilst fewer lymphoedema and hydrocele patients have accessed MMDP services in the same period.

According to the World Health Organization (WHO), MMDP services should be included in the basic primary healthcare package [4–7]. However, progress towards establishing and streamlining MMDP services still remains considerably slow, particularly in the Africa region, which accounts for a considerable proportion of the LF burden [2]. This is evident in the number of countries that have put in place reporting mechanisms for patients with lymphoedema and hydrocele. For instance out of 34 LF endemic countries in the WHO Africa region, only 22 are reporting on lymphoedema patients and 23 on hydrocele patients [2]. Global efforts to eliminate LF through MDA are likely to scale down beyond 2020, and strategic direction of diseases control efforts will most likely pivot towards the provision of MMDP services.

In Zambia, LF is a public health concern as 87 of 118 districts are considered endemic with the prevalence of the circulating filarial antigen above 1.5% [2,8]. MDA for LF was first piloted in Western Province in 2014, and then scaled up nationally in 2015 with annual rounds running until 2018. In 2018, national coverage for MDA was reported at 90.8 % [2]. Morbidity mapping of LF patients has been conducted concurrently with the MDA rounds. Results from the LF mapping exercise have shown that there are many cases of hydrocele and lymphedema spread across all ten provinces in the country. However, there is limited information of what MMDP services are available, where and how LF patients are accessing them. Furthermore, LF patients'

health beliefs and health seeking behaviour towards MMDP services remain largely undocumented in Zambia.

The Zambia Elimination of Neglected Tropical Diseases National Masterplan (2019-2023) places huge emphasis delivery of MMDP services in endemic districts. However, Zambia currently lacks a comprehensive national MMDP strategy for LF or suitable indicators to monitor the provision of MMDP services [8]. As part of efforts to generate evidence to guide the MMDP strategy formulation, the Ministry of Health in collaboration with the University of Zambia, School of Public Health developed a pilot programme using implementation research approaches to identify the most appropriate mechanisms through which MMDP services can be streamlined and integrated into local primary health care systems in Luangwa District to inform potential scale up. This study presents the findings of a preliminary baseline assessment conducted prior to the development and implementation of the pilot programme to understand LF patients' health beliefs and health seeking behaviours towards MMDP services in Zambia. Specifically, this paper reports on the perspectives of health providers, local leaders, community members and patients on their knowledge of LF morbidity, morbidity management practices, health seeking behaviors, and factors affecting access to MMDP services in Luangwa District.

Methods

Study design

An exploratory qualitative research was undertaken as part of a larger baseline formative assessment to inform the development and implementation of an integrated health system intervention to improve access of MMDP services for LF patients in the district. Due to paucity of information within the Zambian context on MDDP, an exploratory qualitative research design was felt to be most appropriate to fully explore community perspectives and common management practices of the most common LF chronic manifestations in the district. The study was conducted between February and April 2019.

Study setting

Luangwa District which is found along the Luangwa River valley in Lusaka Province, has one of the highest prevalence of Circulating Filarial Antigen in Zambia [9]. The district is predominantly rural with an estimated population of 31,665 whose main income generating activities are fishing and farming. The Ministry of Health (MoH) through the local District Health Office is

responsible for primary health care in the district as in other parts of the country. The local population is served by 14 Rural Health Centers and 2 second level Hospitals.

Health systems context

Mass Drug Administration activities which begun in 2015 have predominately been the main recognized health system intervention against LF at district level. While MMDP services are considered as part of the primary health care, local health systems face numerous challenges in their delivery. Very few rural health centers are suitably equipped to provide basic MMDP package of services. Available primary health services are general in nature without specific stand-alone activities for chronic manifestations of LF such as hydrocele and lymphedema. A limited number of health facilities in the area have adequate resources to conduct hydrocelectomies as well as provide lymphoedema management services. In the case of lymphoedema, the most readily available services are pain relief and general health education with IEC materials on display in busy outpatient areas. The health facilities also provide antibiotics to prevent secondary infections as a result of acute attacks as well as lymphatic draining to reduce fluid density. Furthermore, there are existing referral systems is in place, where complicated hydrocele cases are referred to the two hospitals; Katondwe Mission Hospital and Luangwa Boma Hospitals which are equipped to perform hydrocelectomies. Severe cases are referred to the University Teaching Hospital in Lusaka District. Despite surgical interventions being available at health facilities, utilisation has also been reported to be very low. In addition, local healthcare providers and community health workers responsible for conducting case identification and management have limited training on provision of MMDP services.

Participant recruitment

In order to select the most appropriate health facility catchment areas from which data would be collected, the study team conducted a morbidity mapping exercise as available records from the Ministry of Health were not up to date. This mapping exercise consisted a census of patients with the chronic manifestations of LF conducted by Community health workers (CHWs) who are usually engaged by the health facilities in drug distribution during MDA for F campaigns. The CHWs received training on LF case identification and went from household to household recording all patients who exhibited signs of elephantiasis, lymphoedema and hydrocele. There was a total of 237 cases identified during the mapping exercise in the district; 27 lymphedemas, 199 hydroceles and 7 with both hydrocele and lymphedema. Based on the census, eight health facility catchment areas which had the highest number of LF patients were selected as study sites.

These were Luangwa Boma, Mpukha, Katondwe, Kanemela, Chitope, Kasinsa, Mandombe clinics and Luangwa District Hospital. Whilst this study focuses on the formative exploratory qualitative research, the results of the baseline census of LF patients are published elsewhere.

Data collection

Data was collected through a series of focus group discussions (FGDs) and in-depth interviews (IDIs). Interview and FGD guides contained questions on the causes and manifestations of the disease, cultural beliefs, disease management practices, health seeking behavior as well as factors that affect how patients access to healthcare (Finalized tools provided as S1 Appendix). Prior to data collection, the guides/tools were piloted to ensure their suitability by adapting questions where necessary. All FGDs and interviews were audio recorded with the consent of the study participants. In addition, field notes were taken during the course of the interviews. Data collection was done in English and Nyanja to ensure that all participants were able to articulate their perspectives as comprehensively as possible. The recordings were transcribed verbatim and those done in Nyanja were translated to English.

Focus group discussions

There were 4 mixed focus group discussions (2 male and 2 female) held with community members and LF patients in two health facility catchment areas; Luangwa Boma and Mphuka, which had reported the highest number of cases in the district after the morbidity mapping exercise. The participants were conveniently sampled from the communities living in the selected catchment areas. Recruitment was done by the CHWs and health care providers attached to the two health facilities. Participants were invited to take part in the study via telephone by the health facility in-charges. On average each focus group was comprised nine participants aged between 18-50. The focus groups were differentiated by gender due to prevailing cultural beliefs in the district surrounding the ease with which community members could talk about hydrocele which is considered a sensitive topic of discussion. Such a separation encouraged community members to freely express themselves. The FGDs were conducted at the health facility but away from patient areas to ensure the privacy of the participants and avoid interruptions. The FGDs lasted between 1 hour 30 minutes to 2 hours.

In-depth Interviews

A total of twenty-six interviews were also conducted with district neglected tropical disease focal point persons (n=2), community health workers (n=8), health facility staff (n=8) and traditional leaders (n=8). Participants were purposively sampled based on their involvement in the

implementation of LF elimination programmes in the area and were drawn from the 8 health facility catchment areas that had the highest number of LF patients. The interviews with the community leaders were centered around exploring community perspectives of the disease, availability and accessibility of services, stigma and social support structures in their respective communities. Whereas the interviews that were targeted towards the health facility staff, district health officials and CHWs focused on their training to provide MMDP services, knowledge of different management services and factors shaping their ability to effectively provide these services. Participants invited to take part either through telephone calls or face to face. Interviews were conducted at the respective health facilities and the Luangwa district Health Offices. Interviews lasted between 45 minutes to an hour.

Data analysis

A thematic analysis approach was used to analyze the data. In order to determine the different barriers that affect how community members including LF patients access appropriate care, a framework analysis approach was [10]. The study team utilized Levesque et al's conceptual framework on patient centered access to health care which has been extensively used in other studies [7,11,12]. The framework defines access as the "opportunity to reach and obtain appropriate health care services in situations of perceived need for care." It proposes that from a health care provider perspective, access to care can be categorized into five dimensions; approachability, acceptability, availability and accommodation, affordability and appropriateness. Barriers can impact these dimensions which in turn affects an individual's ability to utilize health services. By affecting their ability to perceive, ability to seek, ability to reach, ability to pay and ability to engage with the available health services [1]. These demand and supply side dimensions formed the coding tree from which predetermined themes and sub themes were extracted using both inductive and deductive methods to ensure that existing and emergent themes were exhaustively identified. Preliminary coding of the transcripts was done by PM and AS. JZ reviewed the coding lists to ensure that identified codes were aligned to the to the framework. Subsequently PM, AS and JZ jointly reviewed the codes and developed the final codebook as shown in Table 1.

Table 1. Summary of qualitative coding tree.

**Broader theme**	**Sub theme**
Health beliefs about the chronic stages of LF	Presence of local disease terminologies.
	Causes of infection

	Signs and symptoms of the lymphoedema, hydrocele and acute attacks.
Health seeking behaviour	Consulting traditional healers
	Visits to the health facility
	Knowledge of disease management strategies
Availability and accommodation of health services	Long distances to health facilities
Approachability of health services	Lack of awareness of existing MMDP services
Acceptability of health services	Gender norms
	Social Norms
	Stigmatization
Affordability of health services	Costs of accessing healthcare services

After completion of the data analysis, a stakeholder meeting was held to validate the findings of the study and to confirm that the information captured from the participants was accurately reported. A total of fifty participants took part in the validation meeting including Local Chiefs, traditional leaders, Ministry of Health staff and CHWs. The validation meeting provided an opportunity for further interrogation and alignment of the study findings. More so, it provided a platform to clarify participant perspectives on some of the findings.

Ethical Considerations

Ethical approval was sought from University of Zambia Biomedical Research Ethics Committee (REF.017-11-18) and the National Health Research Authority under the Ministry of Health, Zambia. All participants were informed of the purpose of the assessment, details of study procedures including freedom to withdraw, potential benefits and risks, prior to the commencement of data collection. Thereafter written informed consent was obtained as all participants were 18 years and above.

Results

The study results are presented according to the views of various categories of participants; community members, patients, healthcare providers, community health workers and traditional leaders. Verbatim quotes are also presented to provide context on the participant perspectives. Most of the views on key thematic areas were similar among the different category of participants.

Health beliefs of lymphoedema, hydrocele and elephantiasis.

During the focus group discussions, participants pointed out the local Nyanja names associated with the symptoms of the different manifestations of LF; *tumbu* or *nchofu* for hydrocele and *musakasa* for all forms lymphoedema. The signs and symptoms of the different manifestations of LF chronic conditions were well known by the community members who were able to describe them during the FGDs.

> "This disease of lymphoedema, I used to see people swelling the testicles, legs and to my side at first, I took it to be normal like that's how they were born. All the years I took it like that." (FGD1, Community member)

The most common mentioned causes of LF included eating food that had not been warmed properly, contact with animal faeces, using traditional medicine such as local herbal aphrodisiacs (*mutoto)*, sexual contact with women who were menstruating or who've had a miscarriage, witchcraft, men using pounding sticks after they have been used by women, sitting on chairs which LF patients have used, sitting on stones that women use during food preparation and children vomiting on their mothers during breastfeeding. In addition, there was a belief that the diseases were hereditary. Only a handful of the FGD participants were able to correctly identify mosquitoes as the disease-causing vectors and could be prevented by taking medication.

> "Sleeping with a lady who was pregnant and whose baby dies and they are found in clubs or bars without you knowing anything, you approach her and when she accepts to have sex with her, you end up getting the disease from her." (FGD2, Community member)

> "I didn't know anything I could only see a woman her breast gets swollen, it could be a leg and hand. I thought it was a different disease. I didn't know that the mosquito brings the disease." (FGD1, Community member)

Community actors such as traditional leaders reiterated some of the beliefs that came up during the FGDs. For instance, one of the chiefs stated that;

> "……. Here we believe that if you are passing where there are faeces of animals especially this rain season whether bush animals or village animals and it can be goats, cattle, sheep, pigs, elephants or other animals the disease goes inside the nails making your legs swollen so even if you treat hydrocele it can't be healed and that person will die at that old age with swollen legs like that…." [IDI5, Traditional Leader].

Nevertheless, knowledge of the causes of LF among community actors such as traditional leaders, community-based volunteers and healthcare providers differed greatly depending on their level of involvement in LF disease control activities such as MDA. Most of them acknowledged that they had limited knowledge of how to manage the disease. Healthcare providers who came into regular contact with patients coming to the facility or through community case identification exercises were better at identifying signs and symptoms of lymphoedema, elephantiasis and hydrocele. One health provider indicated;

> "….Even the skin changes and it looks like it doesn't not look to be a normal skin. It changes the color and it becomes hard. For lymphoedema even the limbs, arms legs differ to a normal one. They become different in size….[IDI2, Health provider]."

Healthcare seeking behaviour

It was apparent from the FGDs and interviews that, patients' decision to seek care from both traditional healers and from the health facility was linked to the prevailing ideas about the causes of the disease. Due to the communal belief that LF is hereditary, patients who had seen their family members exhibiting symptoms and not seeking appropriate care did not see the need to go to a health facility. Moreover, community members who viewed it as a disease that arose due to witchcraft rather than mosquitoes were more likely to seek help from traditional healers. There was also a common belief that the remedies provided by the traditional healers were more effective and permanent solutions in comparison to what was provided at the health facilities. One of the participants who had lymphoedema pointed out;

> "I have had three years with this disease and here at this clinic they just give me Panadol when I come so I stopped coming because Panadol doesn't work." (FGD 2, Community member)

The study participants indicated that some lymphoedema patients would first go to a traditional healers and only go to a health facility once their symptoms became more severe. This was because the tattoos and herbs administered by the traditional healers would exacerbate the acute attacks due to LF forcing the patients to go to the health facility for specialised treatment.

> "With traditional beliefs you will think let me go and look for traditional medicine and put traditional tattoos. Now when they put tattoos instead of the legs healing they start swelling because traditional tattoos now start bringing sores because people are different, some of them it just starts swelling without any pain and then you go to see some with doctors or to the clinic." [IDI1, Community Health Worker].

Very few of the community members were able to identify any homebased care strategies that could prevent the progression of lymphoedema and reduce the occurrence of acute attacks. In the case of hydrocele, it was rare for patients to go to health facility to seek interventions such as aspiration of fluid or hydrocelectomies because they were afraid of being rendered infertile, fear of undergoing a surgical procedure or that the surgery would leave them in wheelchairs that made it difficult for them to undertake their regular duties. Some participants narrated that they thought the surgical procedure was actually meant to remove the testicles.

> "Some people are scared to go for operation to say they will be operated, they think when they do they can be gone for good and secondly they say when you are operated you will not have children anymore as a result people go for traditional medicine." (FGD3, Community member)

The patients reported that they were afraid to go the health facility because they felt that their conditions had become so advanced that any treatment they received would not lead to an improvement of their symptoms. Healthcare providers and CHWs pointed out that as a result of this, it was common, for hydrocele patients to come into the health facility with very huge swellings. The loss of hope hampered patient motivation to maintain home based care practices which are critical to ensuring that lymphoedema does not progress to elephantiasis, and that surgical interventions are undertaken early for hydrocele.

> "Say even if am to go to the clinic I won't be healed am already disabled or paralyzed so even if am to go to the clinic I won't be helped, others regardless of the condition being severe, they would remain home and say am disabled already." [IDI3, Community Health Worker].

Health system and cultural barriers to seeking healthcare

Distance to health facility

Luangwa District is very remote and a portion of the district is covered by the Luangwa National Park. As a result, some communities in the district have to travel long distances of up to 20 kilometers and more to the nearest health facility and their access may be inhibited by wildlife attacks such as elephants from the Luangwa National Park. In addition, roads to the health facilities are sometimes impassable and the most common means of transport is bicycles which are inappropriate to transport lymphoedema and hydrocele patients. As such some patients choose to stay home rather than go to the health facility. The district also borders Mozambique and Zimbabwe and patients who may be involved in economic activities which require them to

travel across the borders are often missed out when healthcare providers conduct outreach and follow up visits at community level to provide MMDP services as they may not be found at their homes.

> "Some villages are quite on remote areas. Hence depending on the transportation they may not be able to come from here and also some they come from across our neighboring country across the river in Mozambique." [IDI3, Healthcare Provider].

Lack of awareness of existing MMDP services

A recurring theme across the focus group discussions and interviews is that most community members are not aware that there are MMDP services available at the health facilities or that there are home based care strategies that lymphoedema patients can use to prevent the worsening of their conditions. They indicated that the information that was provided to them by the Community Health Workers was mostly focused on the importance of taking part in annual drug distribution exercises during MDA for LF campaigns. Furthermore, other ongoing community sensitisation exercises tended to focus on diseases such as malaria, HIV/AIDS and maternal, newborn and child related conditions, as LF is not perceived to be a public health priority. Nevertheless, the study participants reported that the healthcare providers and CHWs had now begun conducting community outreach and health talks with the help of LF patients who would act as champions to encourage more community members to utilize MMDP services.

> "What makes these people to come to the clinic, maybe a fellow patient went to the clinic and was assisted then the information is spread to fellows. Then we encourage them that you find you have the right to share with friends that I was helped in this way so that those who are shy can be motivated and go to the clinic openly and express their problems." [IDI3, Community Health Worker].

Costs of accessing healthcare services

Majority of the residents in the district are poor and rely on subsistence farming and fishing for sustenance. As such, when deciding on how best to prioritize available resources, they opted to use the minimal resources they had for food and basic necessities before considering spending on health care, particularly for conditions that they thought were incurable. For this reason, some patients may fear going to the health facility to access MMDP services due to the cost they may have to incur to not only access, but also getting to the health facility considering their disability.

> "Yes, some they think that way. That they will pay for the operation, they think that if doctors refer me to the theatre where am I going to get the money so it's better I just stay with my swollen legs." [IDI5, Traditional Leader].

Despite services at health facilities being free, there is a perception among community members that they are expected to pay fees for treatment services such as hydrocelectomies which dissuades them from seeking care. Furthermore, the opportunity cost associated with taking time off to go to a health facility is not considered preferable. In the event that a patient is found to have a severe case of hydrocele or lymphoedema requiring specialized treatment at one of the three referral facilities, the associated costs such as hiring transportation and out of pocket hospital expenses act as barriers to accessing care. Families that have LF patients are sometimes forced to save money over long periods of time to enable them to access care.

> "So, for them they think, if I start going there with my cost of living, it is difficult to find money, so when they think I start going to the hospital and maybe there are also some payments at the hospital, no its better I just don't go." [IDI1, Community Health Worker].

Gender and Social norms

According to the health care providers, male hydrocele patients are less likely to come to the facility and speak freely about their condition. The participants indicated that one of the most common prevailing cultural belief was that conditions to do with genitalia should not be discussed openly or even shown to members of the opposite sex. As such hydrocele patients are embarrassed to access services and even if they do, they do not easily open up especially if they are being attended to by female health care providers.

> **"P**eople are not open to explain the problem they have especially if a woman is interviewing a man it becomes a big challenge. Others are known that he has such a problem but talking to him, he refuses completely." [IDI2, Community Health Worker].

Nonetheless, having hydrocele was viewed as a marker of high social standing, and men who have it are more likely to be chosen as headmen as they are perceived to be old and wise. This has acted as a deterrent to patients seeking care.

> "…. When they get sick they don't even go to the hospital because they are respected so much. Yes, they respect him a lot even if you reach somewhere he will be the first one to be given the chair just because of what they are seeing, respecting him a lot that one who doesn't have, so mostly if you become a headman they believe you are supposed to have hydrocele

> and you should become the head of the family you are supposed to have that so that even if you this is our leader….."[KII2, Traditional Leader].

Fear of stigmatisation

Fear of stigmatisation also inhibits patients' ability to seek care. During regular case identification exercises by healthcare providers, known hydrocele patients, who are approached for referral to the health facility would either deny having the condition or request to talk to them in private because they are afraid that they would be laughed at if other community members found out that they had hydrocele.

> "Especially if he has hydrocele they start laughing at him so you will find sometimes he can't even be open and be free to go to the hospital because of the fear that people will laugh at me." [KII2, Traditional Leader].

> "They tell you we can talk in a hidden place or maybe you just come later, because there are people present." [IDI1, Community Health Worker].

Discussion

Lymphatic filariasis infection if left untreated results in lymphoedema, elephantiasis and hydrocele which are not only debilitating but without care may result in permanent disability. MMDP services provide an opportunity through which patients could have access to management strategies that ensure their conditions do not progress to more advanced stages allowing them to be fully involved in community activities and undertake personal daily tasks. Various studies have been conducted in Luangwa district exploring the prevalence of the disease [9] and how community engagement strategies are able to shape participation in Mass drug administration campaigns [13], however there have been no studies exploring how the socio-cultural context of the area shapes morbidity management practices at health system and community levels. As such this study sought to explore health beliefs and the health seeking behaviour among communities living in the area in order to inform the development of a MMDP programme that would be implemented in the district.

Local communities in Luangwa district were found to believe that some of the causes of hydrocele and lymphoedema included witchcraft, direct contact with infected individuals or objects they have used as well it being a heritable disease which matches to results from similar studies in other LF endemic countries [14–16]. Moreover, the poor understanding of the connection

between mosquitoes as the cause of filarial infection and the more advanced stages of disease has also been shown elsewhere [14,17,18]. Although a few of the community members were able to correctly identify mosquitoes as the disease-causing vectors and could list some preventive measures such as taking part in mass drug distribution exercises, knowledge of morbidity management practices was very minimal.

As is the case with other low resource settings, community health workers who come into contact with communities are often the main source of information on diseases and management strategies[17]. In the absence of a national MMDP strategy for LF in Zambia which ought to provide guidance on training of CHWs, the inclusion of key health messages related to morbidity management during community sensitization exercises is a missed opportunity. Largely because most of the information that they provide during community mobilization exercises is limited to creating awareness on MDA and the importance of participation in annual rounds.

Despite seeking care from healthcare providers and traditional healers, our study found that traditional healers are still the preferred first line of care for most LF patient living in the community as has been reported elsewhere [14,17,19,20]. Most patients only go to the health facility once their pain has become too severe and their symptoms have progressed. This has a negative bearing on their treatment outcomes. The inclusion of traditional healers in the delivery of morbidity management programmes is an opportunity that can be explored especially given that some of them were found to be LF patients themselves and as such can act as champions. We also found that patient motivation amongst lymphoedema patients often wanes once they realize that their conditions can only be managed over time and are not completely curable. As has been the case in other studies [15,17,20,21]. There were low uptake levels for surgical interventions for hydrocele patients due to fear of the surgery itself, fear of sterility and anticipated costs of surgery. Similar to other studies exploring health seeking behaviour, factors that were found have the most significant effect on accessing MMDP services included cost, lack of awareness of existing services, gender and social norms and stigma [17,19,22,23].

Morbidity management and disability prevention services remain a critical component of the global strategy to address the lymphatic filariasis burden in most low-income settings. Whilst most efforts have predominately been centered around MDA for LF, MMDP services provide a platform to complement these efforts. Studies have documented that creating demand for MMDP services has also had a positive impact on community acceptability of MDA for LF [24]. Morbidity management programmes for lymphedema and hydrocele have been reported to

increase community support for and hence participation in MDA for LF [25]. These programmes provide training on self-management of lymphedema for patients and hydrocele surgical operation for the healthcare providers. Community knowledge of available care, including surgery for hydrocele patients motivated people to participate in MDA for LF. Lymphedema management programmes also provided patients with a platform to share information with other community members about the disease and the benefits of the drugs [26].

Strengths and limitations of the study

This was an exploratory qualitative study that sought to gather data on one of a most neglected, but prevalent public health problem in Zambia. Data were gathered from a varied category of participants with regards to community experiences of LF patients access to healthcare, which enabled for richer understanding and perspective of the topic. Furthermore, the study research team engaged actors from both health systems, community and household level to provide information on some of the underlying issues and multitude of factors at different levels affecting access to MMDP services in Zambia. However, only a small fraction of patients were interviewed as most of their data was captured using a close ended survey that is reported elsewhere. But even with this limitation, the patients as well as other category of participants were able to provide the required information. Another limitation is that sample of Luangwa District may not represent views of other LF endemic areas in Zambia, but still provides a critical learning point in efforts to create national strategy for MMDP services.

Conclusion

This study found that hydrocele and lymphoedema are well known among communities living in Luangwa District. Moreover, the health seeking behaviour among LF patients living in the communities is largely driven by the causes associated with the disease. Ongoing community sensitisation that highlights the linkage between initial infection through mosquitoes and the more advanced disease stages as well as morbidity management practices are necessary. Training guidelines for healthcare providers and community health workers working in endemic districts should equip them with the necessary knowledge that can help them deliver key health messages such as how to conduct home based care for lymphoedema patients and the importance of hydrocele surgery which are necessary for the success of morbidity management programmes. Health education campaigns at community level should also address some of the patients concerns surrounding access to care including reduced patient motivation to maintain lymphoedema management practices and fear of taking up hydrocele surgery. There is also a

need strengthen referral systems to ensure patients not only get appropriate care but that subsequent follow up is made possible. Furthermore, MMDP programmes should include strategies that seek to empower LF patients by ensuring that they get the required information, to access and use the services at the health facilities.

Acknowledgements

We would like to acknowledge Edson Musonda and Frank Shamilimo from the Ministry of Health who facilitated the study process. This work received financial support from the Coalition for Operational Research on Neglected Tropical Diseases (COR-NTD), which is funded at The Task Force for Global Health primarily by the Bill & Melinda Gates Foundation, by the UK aid from the British government, and by the United States Agency for International Development through its Neglected Tropical Diseases Program.

References

1. Kyu HH, Abate D, Abate KH, Abay SM, Abbafati C, Abbasi N, et al. Global, regional, and national disability-adjusted life-years (DALYs) for 359 diseases and injuries and healthy life expectancy (HALE) for 195 countries and territories, 1990–2017: a systematic analysis for the Global Burden of Disease Study 2017. The Lancet. 2018;392: 1859–1922.

2. World Health Organization. Global programme to eliminate lymphatic filariasis: progress report, 2018. Wkly Epidemiol Rec. 2019;94: 457–470.

3. World Health Organization. Lymphatic filariasis: managing morbidity and preventing disability: an aide-mémoire for national programme managers. World Health Organization; 2013. Report No.: 924150529X.

4. Bangert M, Molyneux DH, Lindsay SW, Fitzpatrick C, Engels D. The cross-cutting contribution of the end of neglected tropical diseases to the sustainable development goals. Infect Dis Poverty. 2017;6: 73.

5. Engels D. Neglected tropical diseases in the Sustainable Development Goals. The Lancet. 2016;387: 223–224.

6. Fitzpatrick C, Engels D. Leaving no one behind: a neglected tropical disease indicator and tracers for the Sustainable Development Goals. Int Health. 2016;8: i15–i18.

7. Levesque J-F, Harris MF, Russell G. Patient-centred access to health care: conceptualising access at the interface of health systems and populations. Int J Equity Health. 2013;12: 18.

8. Ministry of Health, Zambia. Elimination of Neglected Tropical Diseases National Masterplan 2019-2023. Lusaka, Zambia; 2019.

9. Mwase ET, Stensgaard A-S, Nsakashalo-Senkwe M, Mubila L, Mwansa J, Songolo P, et al. Mapping the geographical distribution of lymphatic filariasis in Zambia. PLoS Negl Trop Dis. 2014;8: e2714.

10. Ritchie J, Spencer L. Qualitative data analysis for applied policy research. Qual Res Companion. 2002;573: 305–29.

11. Hailemariam M, Fekadu A, Selamu M, Medhin G, Prince M, Hanlon C. Equitable access to integrated primary mental healthcare for people with severe mental disorders in Ethiopia: a formative study. Int J Equity Health. 2016;15: 121.

12. Davy C, Harfield S, McArthur A, Munn Z, Brown A. Access to primary health care services for Indigenous peoples: A framework synthesis. Int J Equity Health. 2016;15: 163.

13. Silumbwe A, Halwindi H, Zulu JM. How community engagement strategies shape participation in mass drug administration programmes for lymphatic filariasis: The case of Luangwa District, Zambia. PLoS Negl Trop Dis. 2019;13: e0007861.

14. Adhikari RK, Sherchand JB, Mishra SR, Ranabhat K, Pokharel A, Devkota P, et al. Health-seeking behaviors and self-care practices of people with filarial lymphoedema in Nepal: a qualitative study. J Trop Med. 2015;2015.

15. Kouassi BL, Barry A, Heitz-Tokpa K, Krauth SJ, Goépogui A, Baldé MS, et al. Perceptions, knowledge, attitudes and practices for the prevention and control of lymphatic filariasis in Conakry, Republic of Guinea. Acta Trop. 2018;179: 109–116.

16. Akogun O, Akogun M, Apake E, Kale O. Rapid community identification, pain and distress associated with lymphoedema and adenolymphangitis due to lymphatic filariasis in resource-limited communities of North-eastern Nigeria. Acta Trop. 2011;120: S62–S68.

17. Person B, Addiss DG, Bartholomew LK, Meijer C, Pou V, Van den Borne B. Health-seeking behaviors and self-care practices of Dominican women with lymphoedema of the leg: implications for lymphoedema management programs. Filaria J. 2006;5: 1–9.

18. Babu BV, Mishra S, Nayak AN. Marriage, sex, and hydrocele: an ethnographic study on the effect of filarial hydrocele on conjugal life and marriageability from Orissa, India. PLoS Negl Trop Dis. 2009;3: e414.

19. Stanton MC, Best A, Cliffe M, Kelly-Hope L, Biritwum N, Batsa L, et al. Situational analysis of lymphatic filariasis morbidity in Ahanta West District of Ghana. Trop Med Int Health. 2016;21: 236–244.

20. Kumari AK, Das L. Issues in delivering morbidity management for lymphatic filariasis elimination: a study in Pondicherry, South India. Sci World J. 2012;2012.

21. Yahathugoda T, Wickramasinghe D, Weerasooriya M, Samarawickrema W. Lymphoedema and its management in cases of lymphatic filariasis: the current situation in three suburbs of Matara, Sri Lanka, before the introduction of a morbidity-control programme. Ann Trop Med Parasitol. 2005;99: 501–510.

22. Perera M, Whitehead M, Molyneux D, Weerasooriya M, Gunatilleke G. Neglected patients with a neglected disease? A qualitative study of lymphatic filariasis. PLoS Negl Trop Dis. 2007;1: e128.

23. Wharton-Smith A, Rassi C, Batisso E, Ortu G, King R, Endriyas M, et al. Gender-related factors affecting health seeking for neglected tropical diseases: findings from a qualitative study in Ethiopia. PLoS Negl Trop Dis. 2019;13: e0007840.

24. Sodahlon YK, Dorkenoo AM, Morgah K, Nabiliou K, Agbo K, Miller R, et al. A success story: Togo is moving toward becoming the first sub-Saharan African nation to eliminate lymphatic filariasis through mass drug administration and countrywide morbidity alleviation. PLoS Negl Trop Dis. 2013;7: e2080.

25. Malecela MN, Lazarus W, Mwingira U, Mwakitalu E, Makene C, Kabali C, et al. Eliminating LF: a progress report from Tanzania. J Lymphoedema. 2009;4: 10–12.

26. Silumbwe A, Zulu JM, Halwindi H, Jacobs C, Zgambo J, Dambe R, et al. A systematic review of factors that shape implementation of mass drug administration for lymphatic filariasis in sub-Saharan Africa. BMC Public Health. 2017;17: 484.

Click here to access/download
Supporting Information
S1 Appendix.docx

Reviewer 1.

1. The authors are presenting the findings of a KAPP (knowledge attitude, practices and perceptions) study on lymphatic filarial disease aetiology and management among three stakeholder groups (patients, community and health providers) in an endemic setting in the district of Luangwa in Zambia where the majority appear to be of low educational attainment with more or less primitive health care facilities. The writing needs to be improved overall so as to improve the clarity of the message conveyed, Results documented were insufficient and erroneous and not presented clearly.

Thank you very much for your feedback. The authors have reviewed the writing and made suitable edits to ensure that the reader experience is improved.

2. I feel that the title " Access to morbidity management and disability prevention services for lymphatic filariasis in Luangwa district, Zambia: A mixed methods study" is somewhat inappropriate as the manuscript does not detail the availability of MMDP health services in the region (number of health facilities that can perform hydrocelectomies, facilities which provide limb care etc.). Without knowing the availability of baseline facilities I feel that you cannot discuss access (defined as "opportunity to reach and obtain appropriate health care services in situations of perceived need for care") to these services. What the authors are describing are the health seeking behavior of patients and factors that influence the behavior patterns.

As part of the baseline assesment, a rapid assessment in 8 of the 16 health facilities in the district and their ability to provide MMDP services was conducted. It was found that very few of them were suitably equipped to provide these services. A brief statement on the availability of services at the health facilities in the area has been included within the description of the study settings of the paper (Lines 150-167). However, we have adjusted the manuscript to primarily reflect community perspective around health seeking behaviour as per the reviewer's suggestion.

3. Overall writing is poor and the meaning of certain statements are rather ambiguous and not clear. This is just one of the instances, Eg, "....of the MMDP intervention such as the design training curriculums, health education and...." .There are many more grammatical errors which need to be attended.

The authors have reviewed the manuscript and paid attention to the concern by the reviewer on grammatical errors and resolved them.

4. The KAPPP findings were interesting but somewhat vague due to lack of quantitative data. I feel that the Results section need to be re-written as presentation of research findings is not very clear and rather confusing as outcomes of all surveys are presented together and discussed in this section. Furthermore the data given in the results section appear to be incorrect. Percentages without the actual values of the variable are unacceptable as there is no way to verify the accuracy of data. Perhaps using tables to present the quantitative data may increase the clarity of the findings. The results of the 3 surveys; patient KAPPP, FGD

and interviews (specify the group, primary health care providers/ Key informants/ community leaders?) if presented separately may be clearer to the reader as well as the authors

Based on the feedback from the reviewers, the authors decided to focus on the qualitative findings and present the quantitative findings as a separate publication. As such the manuscript only present a revised version of the qualitative findings.

5. Is it possible to specify the key areas that were investigated by the KAPP survey, the questions included to the questionnaire ? used on the patients, In the FGDs were the same questions asked from the community was it approached differently? Was the approach similar for all FGDs? With regard to interviews detail the core areas that were covered

We have included a descriptions of the key questions that were asked during the interviews and focus groups as well as added the data collection tools as supplementary file (Lines 184-186 & S1 Appendix).

6. The basic results documented are erroneous and contradictory, Lines 229 & 230; Patient characteristics There were 237 pts.......199 hydrocele, 27 lymphoedema, 7 both lymphoedema and hydrocele, addition brings the total to 233, Line 320 says of the 22 patients with lymphedema? I think even the authors are not clear about how many patients had lymphoedema. The number of lymphoedema patients are rather low to discuss the morbidity management measures practiced by them. Presenting the results as percentages is not acceptable, include the actual value of the variables as well.

As mentioned above, the quantitative component of the assessment has been removed to focus on the qualitative findings from the interviews and the focus group discussions.

7. Line 239, Knowledge of lymphedema, elephantiasis and hydrocele, It is important to document the disease knowledge among patients rather than community, there is no data on this aspect.

The selection of participants for the focus group discussions also included lymphoedema and hydrocele patients to ensure that their perspectives were also represented.

8. With regard to FGD, what was the composition of the sample? (It says a convenient sample) how many patients were included? What was the age range?

The FGDs were comprised of community members aged 18-50 drawn from sampled health facility catchment areas who were able to attend the discussion.

9. Line 354, Social norms. Appear to be contradictory" Having hydrocele is viewed as a marker of high social standing and men who had it are more likely to be chosen as headmen as they are perceived to be old and wise" and the statement "Though LF patients were considered to be in a pitiful state" and lines 376-380 under Stigmatization " Fear of stigmatization also inhibits patients' ability to seek care....would either deny having the condition or request to talk to them in private because they are afraid that they would be laughed at if other community members found out. Are these statement derived from the FGD or are these the authors views?

The statements were derived from the data that was collected, a verbatim quote has been included in the section on stigmatization (Lines 425-429).

10. Beliefs (line 342) were these derived from FGDs or Patient surveys or past publications? (Shawa et al 2013)

All the findings presented are drawn from the data collected during the focus group discussions and the interviews.

11. The manuscript is too lengthy and include a lot of detail on implementation research approaches (lines 102-126) patient access to care frame work guidelines (191-195)but detail (data) on the current study outcomes and the Discussion are inadequate. The authors should compare and discuss the similarities and differences of their results with past reports (publications) on LF in Zimbabwe and elsewhere, discuss the limitations of the study etc.

After revisions based on reviewer's comments, the length of the manuscript has reduced. We have also drawn comparisons with other relevant studies and situated our work within the field.

<u>Reviewer 2.</u>

1. The manuscript is too long given the number of focus groups and in-depth interview conducted. I would suggest the authors to focus on the key findings than listing everything here.

The authors reviewed the manuscript and reviewer's comments and decided to focus specifically on the qualitative findings which have been presented, due to these changes the length of the manuscript has reduced.

2. In the study setting please give clear description of the study setting. When was the LF treatment started in the district? How many health facilities are there in the district? What type of health facilities? How is the function of the health system organized in the district etc.

We have included a description of the local primary health system (Lines 150-167).

3. The discussion part is very brief I would suggest the authors would include, comparing their findings with previous studies, the key limitations of their study. The implication of the current study to programme planning and implementation and some recommendations.

The discussion section of the manuscript has been expanded to factor in similar studies in other settings as well having limitations and recommendations.

<u>Reviewer 3.</u>

1. Overall a reasonably written paper addressing a neglected topic, especially in Zambia. The use of mixed methods is good, however as little more delineation between the different groups would be helpful so it is easier to see who is saying what.

The authors have rewritten the results section and included quotations to indicate sources of information alongside relevant textual descriptions.

2. In the abstract the findings could include more hard data/figures as it is a little vague and the challenges should link to the paper key themes so there is consistency

The authors rewrote the abstract to clearly articulate the major findings from the study.

3. The discussion needs more references supporting the statements.

The authors have reviewed additional references and included relevant ones within the introduction and discussion sections of the manuscript.

4. MMDP - inconsistency in how it is presented i.e. sometime abbreviated but not always. Please check all

We have made revisions to ensure that MMDP is used consistently throughout the manuscript.

5. The authors may want to highlight the economic benefits of surgery as published in PloS recently https://www.ncbi.nlm.nih.gov/pubmed/32210436. This paper also has a number of references that the authors here could use/include to better back some of the statements.

Thank you for this recommendation, the authors have included some of the relevant publications within the revised version.

6. In general there is a lack of references to support the paper

The authors have reviewed additional references and included relevant ones within the introduction and discussion sections of the manuscript.

Click here to access/download
Revised Article with Changes Highlighted
Revised Acess to MMDP Services in Luangwa PM.docx
