# Supplementary material for: Health beliefs and health seeking behavior towards lymphatic filariasis morbidity management and disability prevention services in Luangwa District, Zambia: Community and provider perspectives
Source: PLoS Negl Trop Dis. 2021 Feb 22;15(2):e0009075. doi: 10.1371/journal.pntd.0009075 (PMC7932505; doi:10.1371/journal.pntd.0009075)
Supplement: Attachment — Submitted filename: Response to reviewers comments.docx [file pntd.0009075.s003.docx]

**Reviewer 1.**

1. The authors are presenting the findings of a KAPP (knowledge attitude, practices and perceptions) study on lymphatic filarial disease aetiology and management among three stakeholder groups ( patients, community and health providers) in an endemic setting in the district of Luangwa in Zambia where the majority appear to be of low educational attainment with more or less primitive health care facilities. The writing needs to be improved overall so as to improve the clarity of the message conveyed, Results documented were insufficient and erroneous and not presented clearly.

Thank you very much for your feedback. The authors have reviewed the writing and made suitable edits to ensure that the reader experience is improved.

2. I feel that the title “ Access to morbidity management and disability prevention services for lymphatic filariasis in Luangwa district, Zambia: A mixed methods study” is somewhat inappropriate as the manuscript does not detail the availability of MMDP health services in the region (number of health facilities that can perform hydrocelectomies, facilities which provide limb care etc. ). Without knowing the availability of baseline facilities I feel that you cannot discuss access (defined as “opportunity to reach and obtain appropriate health care services in situations of perceived need for care” ) to these services. What the authors are describing are the health seeking behavior of patients and factors that influence the behavior patterns.

As part of the baseline assesment, a rapid assessment in 8 of the 16 health facilities in the district and their ability to provide MMDP services was conducted. It was found that very few of them were suitably equipped to provide these services. A brief statement on the availability of services at the health facilities in the area has been included within the description of the study settings of the paper (Lines 150-167). However, we have adjusted the manuscript to primarily reflect community perspective around health seeking behaviour as per the reviewer’s suggestion.

3. Overall writing is poor and the meaning of certain statements are rather ambiguous and not clear. This is just one of the instances, Eg, “….of the MMDP intervention such as the design training curriculums, health education and….” .There are many more grammatical errors which need to be attended.

The authors have reviewed the manuscript and paid attention to the concern by the reviewer on grammatical errors and resolved them.

4. The KAPPP findings were interesting but somewhat vague due to lack of quantitative data. I feel that the Results section need to be re-written as presentation of research findings is not very clear and rather confusing as outcomes of all surveys are presented together and discussed in this section. Furthermore the data given in the results section appear to be incorrect. Percentages without the actual values of the variable are unacceptable as there is no way to verify the accuracy of data. Perhaps using tables to present the quantitative data may increase the clarity of the findings. The results of the 3 surveys; patient KAPPP, FGD and interviews ( specify the group, primary health care providers/ Key informants/ community leaders?) if presented separately may be clearer to the reader as well as the authors

Based on the feedback from the reviewers, the authors decided to focus on the qualitative findings and present the quantitative findings as a separate publication. As such the manuscript only present a revised version of the qualitative findings.

5. Is it possible to specify the key areas that were investigated by the KAPP survey, the questions included to the questionnaire ? used on the patients, In the FGDs were the same questions asked from the community was it approached differently? Was the approach similar for all FGDs? With regard to interviews detail the core areas that were covered

We have included a descriptions of the key questions that were asked during the interviews and focus groups as well as added the data collection tools as supplementary file (Lines 184-186 & S1 Appendix).

6. The basic results documented are erroneous and contradictory,
   Lines 229 & 230; Patient characteristics There were 237 pts…….199 hydrocele, 27 lymphoedema, 7 both lymphoedema and hydrocele, addition brings the total to 233, 
   Line 320 says of the 22 patients with lymphedema? I think even the authors are not clear about how many patients had lymphoedema. The number of lymphoedema patients are rather low to discuss the morbidity management measures practiced by them. Presenting the results as percentages is not acceptable, include the actual value of the variables as well.

As mentioned above, the quantitative component of the assessment has been removed to focus on the qualitative findings from the interviews and the focus group discussions.

7. Line 239, Knowledge of lymphedema, elephantiasis and hydrocele,
   It is important to document the disease knowledge among patients rather than community, there is no data on this aspect.

The selection of participants for the focus group discussions also included lymphoedema and hydrocele patients to ensure that their perspectives were also represented.

8. With regard to FGD, what was the composition of the sample? (It says a convenient sample) how many patients were included? What was the age range?

The FGDs were comprised of community members aged 18-50 drawn from sampled health facility catchment areas who were able to attend the discussion.

9. Line 354, Social norms. Appear to be contradictory” Having hydrocele is viewed as a marker of high social standing and men who had it are more likely to be chosen as headmen as they are perceived to be old and wise” and the statement “Though LF patients were considered to be in a pitiful state” and lines 376-380 under Stigmatization ” Fear of stigmatization also inhibits patients’ ability to seek care….would either deny having the condition or request to talk to them in private because they are afraid that they would be laughed at if other community members found out. Are these statement derived from the FGD or are these the authors views?

The statements were derived from the data that was collected, a verbatim quote has been included in the section on stigmatization (Lines 425-429).

10. Beliefs (line 342) were these derived from FGDs or Patient surveys or past publications? (Shawa et al 2013)

All the findings presented are drawn from the data collected during the focus group discussions and the interviews.

11. The manuscript is too lengthy and include a lot of detail on implementation research approaches ( lines 102-126 ) patient access to care frame work guidelines (191-195 )but detail (data) on the current study outcomes and the Discussion are inadequate. The authors should compare and discuss the similarities and differences of their results with past reports (publications) on LF in Zimbabwe and elsewhere, discuss the limitations of the study etc.

After revisions based on reviewer’s comments, the length of the manuscript has reduced. We have also drawn comparisons with other relevant studies and situated our work within the field.

**Reviewer 2.**

1. The manuscript is too long given the number of focus groups and in-depth interview conducted. I would suggest the authors to focus on the key findings than listing everything here.

The authors reviewed the manuscript and reviewer’s comments and decided to focus specifically on the qualitative findings which have been presented, due to these changes the length of the manuscript has reduced.

2. In the study setting please give clear description of the study setting. When was the LF treatment started in the district? How many health facilities are there in the district? What type of health facilities? How is the function of the health system organized in the district etc.

We have included a description of the local primary health system (Lines 150-167).

3. The discussion part is very brief I would suggest the authors would include, comparing their findings with previous studies, the key limitations of their study. The implication of the current study to programme planning and implementation and some recommendations.

The discussion section of the manuscript has been expanded to factor in similar studies in other settings as well having limitations and recommendations.

**Reviewer 3.**

1. Overall a reasonably written paper addressing a neglected topic, especially in Zambia. The use of mixed methods is good, however as little more delineation between the different groups would be helpful so it is easier to see who is saying what.

The authors have rewritten the results section and included quotations to indicate sources of information alongside relevant textual descriptions.

2. In the abstract the findings could include more hard data/figures as it is a little vague and the challenges should link to the paper key themes so there is consistency

The authors rewrote the abstract to clearly articulate the major findings from the study.

3. The discussion needs more references supporting the statements.

The authors have reviewed additional references and included relevant ones within the introduction and discussion sections of the manuscript.

4. MMDP - inconsistency in how it is presented i.e. sometime abbreviated but not always. Please check all

We have made revisions to ensure that MMDP is used consistently throughout the manuscript.

5. The authors may want to highlight the economic benefits of surgery as published in PloS recently https://www.ncbi.nlm.nih.gov/pubmed/32210436. This paper also has a number of references that the authors here could use/include to better back some of the statements.

Thank you for this recommendation, the authors have included some of the relevant publications within the revised version.

6. In general there is a lack of references to support the paper

The authors have reviewed additional references and included relevant ones within the introduction and discussion sections of the manuscript.
