# Supplementary material for: Health beliefs and health seeking behavior towards lymphatic filariasis morbidity management and disability prevention services in Luangwa District, Zambia: Community and provider perspectives
Source: PLoS Negl Trop Dis. 2021 Feb 22;15(2):e0009075. doi: 10.1371/journal.pntd.0009075 (PMC7932505; doi:10.1371/journal.pntd.0009075)
Supplement: Attachment — Submitted filename: Response to reviewers.docx [file pntd.0009075.s005.docx]

**Reviewer 1.**

1. **Abstract, line 36 ; numerical values < than 10 may be better expressed in words; 4-four (circled)**

We have now expressed it in words. Line 36

2. **Line 36; repetition of word ‘interview ‘?**

The additional word has been deleted. Line 36

3. **Line 74; Author Summary “costs of accessing healthcare services” is it actual costs or “perceived costs…?**

We are referring to the perceived cost of accessing health care for LF patients as reported in the interviews and discussions with community members. The sentence has been changed to reflect this. Line 74

4. **Line 90; Background “ 72 endemic countries….” the number is much less with countries reaching the target of elimination. Isn’t this the estimate at the initiation of the GPELF?**

Thank you for this comment. Indeed, we have now updated the number to 49, according to the world health organisation figures. Line 90.

5. **Line 108; The target has been pushed to 2030**

Thank you. We have now changed it from 2020 to 2030. Line 108

6. **Line** 116**; ……..across all ten provinces [ ref]. Include a reference**

A suitable reference has now been included. Line 116

7. **Line 123; …..places huge emphasis on**

We have now added the word on. Line 123

8. **Lines 125-130 This sentence is too long, needs to be rephrased.**

The sentence has been rephrased into three separate sentences 125-131.

9. **Line 149; ……which began in…./… begun in …**

The word began has now been replaced with begun. Line 149

10. **Lines 159 &160; What are the challenges faced in delivering the basic MMDP package for lymphoedema?
    Regarding “Very few health facilities are suitably equipped to provide basic MMDP package…. “ Basic lymphoedema management (which is irreversible if it progresses) does not require sophisticated equipment …Needs clean water soap, basin, antibiotic medication, bandages etc. On the other hand, hydrocele requires surgery (need equipment) but the condition can be reversed by surgery.**

Thank you for this comment. Indeed, we agree that the basic MMDP package does not require sophisticated equipment to manage the LF chronic diseases. We have now rephrased the sentence to state that very few health facilities prioritize provision of the basic package of MMDP services. Further, according to the WHO Minimum package of care for the chronic stages of Lymphatic filariasis, patients should have access to surgery for hydrocele. Also, there should be treatment and prevention options to manage acute attacks amongst patients with lymphoedema and elephantiasis. Our study found that a majority of the health facilities in the area did not have adequate medication necessary to treat acute attacks or antifilarials. They also did not have the capacity to perform hydrocele surgery. Lines 149-174

11. **Line 180; ……“ F campaign”… expand**

This was supposed to be LF to represent Lymphatic filariasis. Line 180

12. **Regarding Data collection; specify who did the interviews**

We have included a statement of who conducted the interviews. Lines 198 -200

13. **Line 214;….. Health facility in-charges; Is it staff in-charge of the health facility**

The health facility in-charge does indeed refer to the person who is in charge of the health facility. Depending on the type of facility it could be a nurse, a doctor or a clinical officer.

14. **Line 234; Participants were invited….**

We have included the word were to the sentence. Line 234

15. **Line 240 Sentence is incomplete**

The word utilized has been added to complete the sentence. Line 240

16. **Long distance to health facility- does it fall under approachability?**

Thank you for this comment. We have modified the coding tree to show how the themes relate to one another as well as the story being told in the results section. Check table 1, line 247. Long distance is now shown to explicitly fall under the health systems barriers as explained in the results narrative.

17. **Similarly Lack of awareness- availability and accommodation?**

Thank you for this comment. We have modified the coding tree to show how the themes relate to one another as well as the story being told in the results section. Check table 1, line 247. Lack of awareness is now shown to explicitly fall under the health systems barriers as explained in the results narrative.

18. **Line 389; CHW?**

CHWs is the abbreviation for Community Health Workers. This has been defined when first used in the manuscript in line 179.

**Lines 473-478; needs to be re-phrased as meaning is unclear.**

The sentence has been rephrased. 473-478

19. **Lines 480;…….LF patients……**

We have changed patient to patients. Line 480

20. **Line 495; ….predominantly….**

We have put in the correct spelling of the word predominantly. Line 495

21. **Conclusions:** According to the study outcomes an urgent requirement is the establishment of a properly planned health education program in order to dispel false beliefs on disease causation and more importantly the correct measures that need to implement to prevent disease progression in the case of lymphedema.

Thank you for the comment. we have edited the conclusions and discussed all the deficiencies and potential solutions in the redone discussion section.

22. **Line 574; these are” perceived causes” “…….causes perceived to be associated with the disease”**

We have added the word perceived. Line 574.

23. **Line 581; HE should address issues regarding availability of care free-of-charge, fear of side effects etc.**
    We have expanded the sentence to reflect the reviewer’s suggestions. 583-585
24. **Line 587; “empower and motivate LF patients and community to adhere to skin hygiene based self-care-regime” …?**

We have expanded the sentence to reflect the reviewer’s suggestions. Lines **587-600**

25. **The conclusion of the study is incomplete, suggest expansion focusing on deficiencies and how to rectify these?**

Thank you for the comment. We have edited the conclusions and discussed all the deficiencies and potential solutions.

26. **The limitations of the study design need to be discussed.**

Thank you for this comment the limitations of the study design are discussed in the subsection strengths and limitations. We acknowledge limitations in the sampling approach as we as implications for our findings in other settings. further, we acknowledge that we could have done more interviews with LF patients.

27. **Public health relevance is discussed but there is room for expansion**

The discussion has been modified to clearly highlight the public health relevance of the findings, particularly with regards to informing future country morbidity and disability prevention strategies.

**Reviewer 2.**

1. **It would be better to mention how they determined the number of FGDs and in-depth interviews. Was it based on saturation of the data?**

Thanks, we have now clarified that we determined the number of FGDs and IDIs using the principle of data saturation in qualitative research. Line 176-176

2. **Table 1 Summary of qualitative coding tree, would be better if it is presented on an actual tree how showing how each of the sub-themes relate one another. How is the perception affecting the service uptake in addition to the clear direct barriers such as cost and service availability**?

Thank you for this comment. We have modified the coding tree to show how the themes relate to one another, as well as the story being told in the results section. Check table 1, line 247.

3. **Line 224 the statement ‘framework analysis approach was’ is incomplete.**

The word utilized has been added to complete the sentence.

4. **Line 20 and 21: These authors contributed equally to this work is not clear, why the authors wanted to have two of them?**

We have changed the author designation to reflect that all authors contributed equally to the work.

5. **Nonetheless, strengthening the discussion of the public health relevance of the study would be important**

The discussion has been modified to clearly highlight the public health relevance of the findings, particularly with regards to informing future country morbidity and disability prevention strategies.

**Additional edits**

1. We have edited and standardized the grammar across the entire manuscript, including revisions of misspellings and long sentences
2. The title has been modified to reflect the objective and the story presented in the results
